# Identification of a migrasome-related lncRNA signature and its prognostic and immunological role in bladder cancer

**DOI:** 10.3389/fimmu.2026.1763443

**Published:** 2026-04-16

**Authors:** Junlin Shen, Chun Wang, Tian’en Li, Heyang Guan, Mingpeng Zhang, Ruitao Li, Yaqun Zhang, Liwei Liu, Jing Tian, Zhiqun Shang

**Affiliations:** 1 Department of Urology, Tianjin Institute of Urology, The Second Hospital of Tianjin Medical University, Tianjin, China; 2 Haihe Laboratory of Synthetic Biology, Tianjin, China; 3 Department of Thoracic Surgery, Tongji Hospital, Tongji Medical College, HuazhongUniversity of Science and Technology, Wuhan, Hubei, China

**Keywords:** bladder cancer, genomic mutation burden, migrasome-related lncRNAs, prognostic biomarker signature, single-cell transcriptomic profiling, therapeutic sensitivity, tumor immune landscape

## Abstract

**Background:**

Bladder cancer (BLCA) is a highly heterogeneous malignancy with considerable variability in clinical outcomes. Reliable biomarkers for prognosis and treatment guidance remain urgently needed. Migrasomes, recently identified cellular organelles involved in intercellular communication, have been implicated in cancer progression, yet their associated long non-coding RNAs (lncRNAs) and prognostic relevance in BLCA remain unclear.

**Methods:**

We integrated transcriptomic and clinical data from The Cancer Genome Atlas (TCGA) to identify migrasome-related lncRNAs through co-expression analysis. A prognostic signature was constructed using univariate Cox, LASSO, and multivariate regression analyses. Patients were stratified into high- and low-risk groups across training, validation, and entire cohorts. Kaplan–Meier survival, ROC curves, PCA, and Cox analyses were used for validation. Functional annotation was conducted via GO, KEGG, and GSEA. Immune infiltration and tumor microenvironment features were evaluated using ESTIMATE and CIBERSORT, while tumor mutation burden (TMB), immune escape potential, and drug sensitivity were also analyzed. Single-cell RNA sequencing (scRNA-seq) analysis delineated cellular heterogeneity and intercellular communication patterns related to the risk signature in the BLCA microenvironment. Finally, experimental validation of SEC24B-AS1, a key lncRNA within the model, was performed through qRT-PCR, CCK-8, colony formation, and migration assays to confirm its biological relevance.

**Results:**

A robust prognostic signature comprising migrasome-related lncRNAs was established, effectively distinguishing patients into high- and low-risk groups with significantly different survival outcomes. The signature remained an independent prognostic factor after multivariate adjustment. Functional enrichment analyses revealed marked biological distinctions between the two groups. Immune profiling showed higher immune and stromal scores in high-risk patients, together with distinct immune cell composition and function. High-risk tumors also exhibited elevated TMB and increased immune evasion potential. Drug sensitivity analysis identified compounds with differential responses between risk subgroups. Single-cell transcriptomic analysis indicated that the signature reflects cellular heterogeneity and intercellular communication patterns within the BLCA microenvironment. Experimental validation of SEC24B-AS1, a key lncRNA in the model, demonstrated its impact on tumor cell proliferation and migration, supporting the biological relevance of the prognostic signature in BLCA.

**Conclusion:**

This study presents a novel migrasome-related lncRNA signature that independently predicts survival in bladder cancer and reflects the tumor immune landscape.

## 1 Introduction

Bladder cancer (BLCA) remains one of the most frequently diagnosed malignancies of the urinary system, with urothelial carcinoma constituting the predominant histological subtype, accounting for over 90% of cases (1). Despite improvements in early detection and advancements in therapeutic strategies, the prognosis for patients with advanced or metastatic BCa remains unsatisfactory, largely due to its aggressive behavior and high recurrence rates (2). Therefore, the identification of novel prognostic biomarkers and the development of reliable risk assessment models are urgently needed to enhance individualized treatment approaches and improve clinical outcomes.

Migrasomes, a recently characterized class of extracellular vesicle-like organelles, are generated during cellular migration processes (3). These structures arise at the tips or intersections of retraction fibers, with their formation dependent on actin polymerization and cellular movement (3). Migrasomes are capable of packaging and transferring cytoplasmic contents, thereby mediating intercellular communication. Emerging evidence suggests that migrasomes serve as important conveyors of proteins, RNAs, and other bioactive molecules, participating in a variety of physiological processes including tissue development, immune regulation, and cancer metastasis (4–6). Migrasomes are vesicle-like structures that form on retraction fibers during cell migration, with their biogenesis driven by the assembly of tetraspanin- and cholesterol-enriched membrane macrodomains. Experimental and theoretical evidence demonstrates that elevated membrane stiffness, induced by tetraspanin 4 and cholesterol, is essential for migrasome formation (7). Pancreatic cancer cells are capable of generating migrasomes (PCDMs) that are enriched with immunosuppressive factors and actively contribute to the formation of a tumor-promoting microenvironment. By inducing M2-like polarization of macrophages and suppressing T cell activation, PCDMs play a critical role in facilitating pancreatic cancer progression (8). These findings underscore the potential significance of migrasomes in tumor biology and highlight their role in modulating disease progression.

Long non-coding RNAs (lncRNAs), transcripts longer than 200 nucleotides without protein-coding potential, have emerged as critical regulators in oncogenesis (9). They exert their effects through diverse mechanisms, including transcriptional and post-transcriptional regulation, as well as epigenetic modifications (10). In bladder cancer, accumulating evidence indicates that specific lncRNAs are involved in key cellular processes such as proliferation, apoptosis, migration, and immune evasio (11). Certain lncRNAs have also been identified as promising diagnostic and prognostic biomarkers, offering new avenues for therapeutic intervention (12, 13). Nevertheless, the functional interplay between migrasomes and lncRNAs in BLCA remains largely unexplored.

Currently, clinical risk stratification for BLCA primarily relies on clinicopathological features such as TNM staging and histological grading. However, a major unmet clinical need is that patients within the same clinical stage frequently exhibit highly variable clinical trajectories and distinct responses to therapies, largely due to underlying molecular and genetic heterogeneity. Traditional staging systems are predominantly anatomically based and fail to capture the complex tumor microenvironment or predict therapeutic vulnerabilities. Therefore, the development of novel molecular prognostic signatures is urgently required. Such signatures could complement conventional clinical parameters by bridging the gap between survival prediction and personalized therapeutic guidance, particularly in the era of targeted and immunotherapies.

Given the critical roles of both migrasomes and lncRNAs in tumorigenesis, investigating migrasome-associated lncRNAs may provide novel insights into BLCA progression and patient stratification. Biologically, migrasomes can package and release cytosolic contents to mediate intercellular communication. Given that bladder cancer is a highly invasive malignancy fundamentally driven by cell migration—a process strictly coupled with migrasome biogenesis—investigating migrasome-related lncRNAs offers a highly rational approach to predicting patient prognosis. In this study, we aimed to establish a prognostic model based on migrasome-related lncRNAs in bladder cancer. By categorizing patients into distinct risk groups, our model seeks to predict survival outcomes and inform clinical decision-making. Furthermore, our findings shed light on the biological significance of migrasome-associated lncRNAs and their potential as therapeutic targets.

## 2 Methods

### 2.1 Acquisition of transcriptomic and clinical data for bladder cancer

Transcriptomic profiles and corresponding clinical data for bladder cancer (BLCA) were obtained from The Cancer Genome Atlas (TCGA) database to enable an integrated analysis of gene expression patterns and clinical outcomes. A total of 431 RNA sequencing (RNA-seq) datasets were retrieved, comprising 412 tumor samples and 19 adjacent normal tissue samples. Matched clinical information was available for 412 patients. All datasets were accessed via the Genomic Data Commons (GDC) Data Portal (https://portal.gdc.cancer.gov), ensuring standardized data processing and quality control across samples.

### 2.2 Identification and development of a migrasome-associated lncRNA prognostic model

To explore prognostically relevant migrasome-associated long non-coding RNAs (lncRNAs) in BLCA, transcriptomic profiles were first processed using the “limma” package in R. Candidate lncRNAs were selected based on Pearson correlation analysis with known migrasome-related genes, using a stringent cutoff of |R| > 0.4 and p < 0.001. Expression levels of the filtered lncRNAs were extracted for further analysis. To visualize the regulatory relationships, Sankey diagrams were generated with the “ggalluvial” package, depicting the linkage between migrasome-related genes and the identified lncRNAs.

The cohort was then randomly partitioned into a training cohort and a validation cohort to construct and internally validate the risk model. In the training cohort, univariate Cox proportional hazards regression was performed to screen lncRNAs significantly associated with overall survival (p < 0.05). Lasso (Least Absolute Shrinkage and Selection Operator) regression was subsequently applied to minimize overfitting and refine the feature set, with the optimal λ parameter.

Finally, multivariate Cox regression analysis was conducted using the lncRNAs retained by Lasso, resulting in the construction of a robust prognostic model. A stepwise regression approach was employed to further optimize the model, ensuring both parsimony and predictive accuracy.

### 2.3 Correlation assessment between migrasome-associated genes and prognostic lncRNAs

To explore potential regulatory relationships, we assessed the correlations between migrasome-related genes and the lncRNAs constituting the prognostic signature. Expression matrices for both gene sets were extracted from the BLCA transcriptomic data. Pearson correlation analysis was conducted for each migrasome gene–lncRNA pair, and correlation coefficients along with corresponding p-values were calculated. Statistical significance was categorized as ***p < 0.001, **p < 0.01, and *p < 0.05. The resulting correlation patterns were visualized using heatmaps, where color gradients were applied to represent the strength and direction of the correlations.

### 2.4 Survival analysis based on the migrasome-associated lncRNA signature

To evaluate the prognostic relevance of the migrasome-associated lncRNA signature, Kaplan-Meier survival analyses were performed for both overall survival (OS) and progression-free survival (PFS). Patients were stratified into high- and low-risk groups according to the calculated risk scores. Survival curves for OS were separately plotted for the training cohort, validation cohort, and the entire cohort. PFS analysis was carried out based on clinical follow-up information. Differences between groups were assessed using the log-rank test, with statistical significance defined as p < 0.001.

### 2.5 Risk stratification analysis based on the migrasome-associated lncRNA signature

To further illustrate the prognostic utility of the migrasome-associated lncRNA signature, risk stratification analyses were conducted across the training, validation, and entire cohorts. Within each cohort, patients were ranked according to their calculated risk scores and subsequently divided into high- and low-risk groups based on the median cutoff. Risk distribution plots were generated to depict the spread of risk scores, with a dashed line marking the boundary between the two groups. Additionally, survival status scatter plots were produced, where each point represents an individual patient; red dots indicate deceased cases, while green dots represent surviving individuals. Heatmaps were constructed to visualize the expression profiles of the signature lncRNAs across risk groups, with data normalized by row and annotated according to risk classification.

### 2.6 Independent prognostic evaluation via Cox regression

To determine whether the migrasome-associated lncRNA risk score serves as an independent prognostic indicator, univariate and multivariate Cox proportional hazards analyses were conducted. Variables assessed in the univariate analysis included age, tumor grade, clinical stage, and the calculated risk score. Parameters showing statistical significance (p < 0.05) were subsequently incorporated into the multivariate Cox regression to control for confounding effects. For each variable, hazard ratios (HR), 95% confidence intervals (CI), and p-values were calculated and reported.

### 2.7 ROC curve analysis for predictive performance

Time-dependent receiver operating characteristic (ROC) curves were constructed to evaluate the predictive accuracy of the lncRNA-based prognostic signature at 1-, 3-, and 5-year time points. The area under the curve (AUC) was used as a measure of model performance. To assess the robustness of the signature relative to conventional clinical variables, its 5-year AUC was compared against age, sex, tumor grade, and clinical stage.

### 2.8 Model evaluation via concordance index and nomogram construction

To assess the predictive reliability and clinical applicability of the migrasome-associated lncRNA signature, both concordance index (C-index) analysis and nomogram construction were performed.

First, univariate Cox proportional hazards models were constructed for the lncRNA-based risk score and key clinical variables, including age, gender, grade, and stage. The `cph` function from the rms package was used to fit survival models, and the `cindex` function from the *pec* package was applied to compute time-dependent C-index values across a 10-year interval. Bootstrap cross-validation with 1,000 iterations was used to ensure model robustness. Comparative performance was visualized by plotting C-index trajectories for each variable over time.

To enhance clinical interpretability, a nomogram was built integrating the risk score and clinical parameters using the *regplot* package. The multivariate Cox model was trained using the `coxph` function, and the nomogram included graphical components such as density plots and score boxes to visualize individual risk contributions. The model’s predictive capability was evaluated at 1-, 3-, and 5-year time points.

Calibration curves were generated to validate the nomogram’s performance. Using the calibrate function from the rms package, calibration plots were produced based on Kaplan-Meier survival estimates and 1,000 bootstrap replicates for each time point. The observed versus predicted survival probabilities were compared at 1, 3, and 5 years. Consistency between predicted and actual outcomes indicated the model’s calibration accuracy.

### 2.9 Principal component analysis

Principal component analysis (PCA) was carried out to visualize the distribution patterns of patients in the high- and low-risk groups. Four gene expression matrices were analyzed (1): all genes (2), migrasome-related genes, (3) migrasome-associated lncRNAs, and (4) signature lncRNAs. Data were preprocessed by removing low-expression genes and applying log-transformation. Normal tissue samples were excluded based on TCGA barcodes. PCA was implemented using the `prcomp` function with scaling, and results were visualized in three-dimensional space via the scatterplot3d package, with risk groups indicated by color coding.

### 2.10 Functional enrichment analysis of differentially expressed genes

Gene Ontology (GO) enrichment analysis was performed on differentially expressed genes (DEGs), covering biological processes (BP), cellular components (CC), and molecular functions (MF). The enrichGO function from the *clusterProfiler* package was used, with p < 0.05 and q < 0.05 set as significance thresholds. Top 10 enriched terms for each GO category were visualized using bar plots, and a circular plot highlighted the proportion and significance of enriched genes across functional terms. Kyoto Encyclopedia of Genes and Genomes (KEGG) pathway analysis was also conducted on DEGs using the enrichKEGG function, applying the same thresholds (p < 0.05, q < 0.05). Enriched pathways were illustrated in bar plots to highlight key signaling processes related to immune response, cell communication, and tissue structure.

### 2.11 Gene set enrichment analysis of high- and low-risk groups

To explore functional differences between high- and low-risk patients, Gene Set Enrichment Analysis (GSEA) was conducted. After calculating log fold changes in gene expression between the two groups, GSEA was performed using curated KEGG pathway gene sets. Enriched pathways were filtered using a p-value cutoff of 0.05. The top five significantly enriched pathways in each risk group were visualized to highlight distinct biological processes associated with tumor progression and immune modulation.

### 2.12 Acquisition of somatic mutation data

Somatic mutation profiles for BLCA were retrieved from the TCGA GDC data portal. The mutation annotation format (MAF) files were downloaded and preprocessed to include only tumor samples. These data provided a foundation for subsequent mutational landscape analyses, including tumor mutational burden (TMB) calculation and mutation frequency comparisons across risk groups.

### 2.13 Tumor microenvironment and immune infiltration analysis

The tumor microenvironment (TME) was assessed by calculating stromal, immune, and ESTIMATE scores using the ESTIMATE algorithm. Differences in TME scores between high- and low-risk groups were visualized using violin plots overlaid with boxplots. Statistical comparisons were made using the Wilcoxon test.

Immune cell infiltration was evaluated using CIBERSORT, which estimates the relative proportions of 22 immune cell types. Samples with CIBERSORT p-values < 0.05 were retained for downstream analysis. Group-wise comparisons were displayed via stacked bar plots for global distribution and boxplots for cell types with statistically significant differences.

To further assess immune functional states, single-sample GSEA (ssGSEA) was applied using immune-related gene sets. Immune activity scores for each sample were calculated and compared between groups, with the results presented as boxplots.

### 2.14 Tumor mutational burden analysis

TMB was computed as the number of non-synonymous mutations per megabase of the coding genome. The 15 most frequently mutated genes in BLCA were identified, and their mutation patterns were visualized using the `oncoplot` function from the *maftools* package. High- and low-risk cohorts were analyzed separately.

To assess the prognostic impact of TMB in conjunction with the risk score, patients were categorized into four subgroups: high-TMB + high-risk, high-TMB + low-risk, low-TMB + high-risk, and low-TMB + low-risk. Kaplan-Meier survival curves were plotted using the ‘ggsurvplot’ function from *survminer*.

In addition, tumor immune dysfunction and exclusion (TIDE) scores were extracted and compared between risk groups to estimate immune evasion potential.

### 2.15 Drug sensitivity analysis

To investigate differences in therapeutic response, drug sensitivity data were analyzed between high- and low-risk groups. Estimated drug response (IC50) values were log-transformed and compared using the Wilcoxon test. Only drugs with statistically significant differences (p < 0.001) were visualized. Boxplots were used to display the sensitivity distribution of selected drugs across the two risk groups.

### 2.16 Processing of single-cell RNA sequencing data

Single-cell RNA sequencing (scRNA-seq) datasets related to bladder cancer were downloaded from the Gene Expression Omnibus (GEO) database, including eight samples (GSM8273655, GSM8273657, GSM8273672, GSM8273675, GSM3729178, GSM3729179, GSM8273661, and GSM8273665). Raw count matrices generated using the 10X Genomics platform were imported into R and analyzed with the “Seurat” package. The analytical pipeline comprised several major steps: cell quality control, elimination of doublets, normalization, data integration, and subsequent downstream analyses. During preprocessing, low-quality cells were excluded if they expressed fewer than 300 genes, contained under 1,000 total UMI counts, or exhibited mitochondrial gene expression exceeding 10% of total transcripts. Doublet detection and removal were carried out using the “DoubletFinder” package, assuming an expected doublet rate of approximately 7.5–8.0%. Following filtration and normalization, all samples were merged, and Harmony was employed to mitigate inter-sample batch variation. Dimensional reduction was subsequently performed via principal component analysis (PCA), and a low-dimensional embedding was generated using Uniform Manifold Approximation and Projection (UMAP) based on the top 20 Harmony components. Cluster annotation was conducted through manual inspection of canonical marker genes obtained with the “FindAllMarkers” function. The identified cell populations were characterized as urothelial tumor epithelial cells, tumor-associated macrophages (TAMs), cytotoxic T/NK cells, neuroendocrine-like tumor cells, cycling tumor cells, endothelial cells, cancer-associated fibroblasts (CAFs), pericytes, keratinized urothelial cells, metabolically active urothelial epithelial cells, conventional dendritic cells type 2 (cDC2), and plasma cells. For evaluating the single-cell distribution of the lncRNA-based prognostic signature, each cell was assigned a risk value computed as a weighted sum of the normalized expression levels of six selected lncRNAs, with weights derived from Cox regression coefficients. Cells with non-zero risk values were divided into high- and low-risk subgroups using the median score as the cutoff. Variations in cellular composition between these groups were assessed and visualized with stacked bar charts, whereas the spatial expression of the individual lncRNAs was illustrated through “FeaturePlot” and related visualization functions.

To further characterize intercellular communication patterns, we implemented the “CellChat” framework. Cell–cell interaction probabilities were inferred from normalized transcriptomic profiles and predefined cell-type identities. Enriched signaling pathways were identified and depicted using circular network diagrams, chord plots, and heatmaps to delineate pathway-level communication differences associated with the risk stratification.

### 2.17 Cell culture and transfection

The human bladder cancer cell lines UM-UC3 and T24 were obtained from the Cell Bank of the Chinese Academy of Sciences (Shanghai, China). UM-UC3 cells were maintained in Dulbecco’s DMEM(VivaCell, China), while T24 cells were cultured in RPMI-1640 medium (VivaCell, China), each supplemented with 10% fetal bovine serum (ExCell Bio, China). All cells were incubated at 37 °C in a humidified atmosphere containing 5% CO_2_. The use of these commercially acquired cell lines complied with institutional and national ethical guidelines. Transient silencing of SEC24B-AS1 was carried out using small interfering RNAs synthesized by JTSBIO Co. (Wuhan, China). Transfection procedures were performed with Lipofectamine™ 3000 reagent (Invitrogen, USA) according to the manufacturer’s protocol. The specific siRNA sequences employed for transfection are listed in **Supplementary Table 1**.

### 2.18 Quantitative real-time PCR

Total RNA was isolated from cultured cells using Trizol reagent (Invitrogen, USA) in accordance with the manufacturer’s guidelines. Complementary DNA (cDNA) was synthesized from the extracted RNA with a reverse transcription kit (Thermo Scientific, USA). Quantitative real-time PCR was subsequently carried out using SYBR Green Master Mix (Roche) on a real-time PCR detection system to measure gene expression levels. The primer sequences utilized for amplification are provided in **Supplementary Table 1**.

### 2.19 CCK-8 assay

UM-UC3 and T24 cells were seeded into 96-well plates at an initial density of approximately 1 × 10³ cells per well. After cell attachment, 10 μL of CCK-8 reagent was added to each well at the indicated time points, followed by incubation at 37 °C for 1 hour. The absorbance at 450 nm was then measured using an enzymatic calibrator to quantify cellular proliferation and viability.

### 2.20 Colony formation assay

To assess clonogenic capacity, UM-UC3 and T24 bladder cancer cells were plated in six-well culture dishes at a density of 500 cells per well. Cells were maintained in complete medium (DMEM or RPMI-1640 supplemented with 10% fetal bovine serum. The medium was refreshed periodically, and cultures were allowed to grow for approximately 14 days until discrete colonies became visible to the naked eye. Subsequently, the colonies were rinsed gently with phosphate-buffered saline (PBS), fixed with 4% paraformaldehyde for 15 minutes, and stained using crystal violet solution. Images of the stained colonies were captured and enumerated for quantitative analysis.

### 2.21 Cell migration assay

Cell migratory ability was evaluated using Transwell chambers (Corning Costar, USA) with an 8-μm pore membrane. The lower chambers were filled with complete medium containing 10% FBS to act as a chemoattractant, whereas 1 × 10^4^ cells suspended in serum-free medium were added to the upper chambers. Following 48 hours of incubation, non-migrated cells on the upper surface were carefully removed, and migrated cells adhering to the lower surface were fixed, stained with crystal violet, and imaged under a light microscope. ImageJ software was used to quantify the migrated cells.

### 2.22 Wound-healing assay

UM-UC3 and T24 cells were grown in six-well plates until they reached near-complete confluence. A linear scratch was introduced across the monolayer using a sterile 200 μL pipette tip to mimic wound formation. After removing detached cells by rinsing twice with PBS, the remaining adherent cells were maintained in serum-free medium. Images of the initial wound were captured under an inverted microscope at 10× magnification. After 24 hours of incubation, the same fields were re-imaged, and wound closure was quantified using *ImageJ* software to calculate the percentage of migration.

### 2.23 Western blot

Total protein was extracted using RIPA lysis buffer supplemented with PMSF (1:100). Following a 30-min incubation on ice and subsequent sonication, the lysates were subjected to centrifugation at 12,000 × g (4 °C) for 20 min to harvest the supernatants. Protein concentrations were evaluated via a BCA assay kit. Next, 20 μg of protein aliquots were resolved on 10% SDS-PAGE gels and electroblotted onto PVDF membranes. To prevent non-specific binding, the membranes were blocked with 5% skim milk prepared in TBST for 1 h at room temperature. The blots were then probed overnight at 4 °C with primary antibodies against ITGA5 (1:1000, 10569-1-AP, Proteintech, China), TSPAN4 (1:1000, A10253, Abclonal, China), and GAPDH (1:50000, 60004-1-Ig, Proteintech, China). After washing with TBST three times, appropriate secondary antibodies were applied for 1 h at room temperature. Finally, following three additional TBST washes, the targeted protein bands were visualized.

### 2.24 Statistical analysis

All statistical analyses and graphical visualizations were performed using R software and its associated packages and GraphPad Prism. Pearson correlation analysis was utilized to evaluate the relationships between migrasome-related genes and lncRNAs. To assess baseline clinical characteristic differences between the high- and low-risk groups, Chi-square tests were employed for categorical variables. For comparisons of continuous variables, such as tumor microenvironment scores and estimated drug response (IC50) values, the non-parametric Wilcoxon test was applied. Survival analyses were conducted using the Kaplan-Meier method, and survival differences between groups were compared using the log-rank test. To identify independent prognostic factors and construct the risk signature, univariate Cox proportional hazards regression, LASSO regression with cross-validation, multivariate Cox regression, and stepwise regression were sequentially performed. The predictive performance of the model was evaluated using time-dependent receiver operating characteristic (ROC) curves, area under the curve (AUC) values, and the concordance index (C-index) internally validated via bootstrap resampling. For functional enrichment analyses, significance was defined using both *p*-values and *q*-values (*q* < 0.05) to control for the false discovery rate (FDR) during multiple testing. For *in vitro* functional experiments, the Shapiro-Wilk test was performed to assess the normality of these experimental data. For data meeting normal distribution, the F-test was utilized to confirm the homogeneity of variances, followed by the parametric independent Student’s t-test to determine statistical significance between the control and knockdown groups. Throughout the study, statistical significance was categorized as *p < 0.05, **p < 0.01, and ***p < 0.001.

## 3 Results

### 3.1 Identification and integration of migrasome-related lncRNAs into a prognostic signature for bladder cancer

To identify genes functionally linked to migrasomes, we first conducted a comprehensive literature review, complemented by database mining using GeneCards. In the GeneCards query, genes with a Relevance Score greater than 1 were retained. This strategy yielded a list of eleven migrasome-related genes, including TSPAN4, NDST1, PKD1, CPQ, PKD2, EPCIP, EOGT, TMX2-CTNND1, PIGK, ITGA5 and ITGB1 (6, 8, 14, 15).

**Figure 1:**
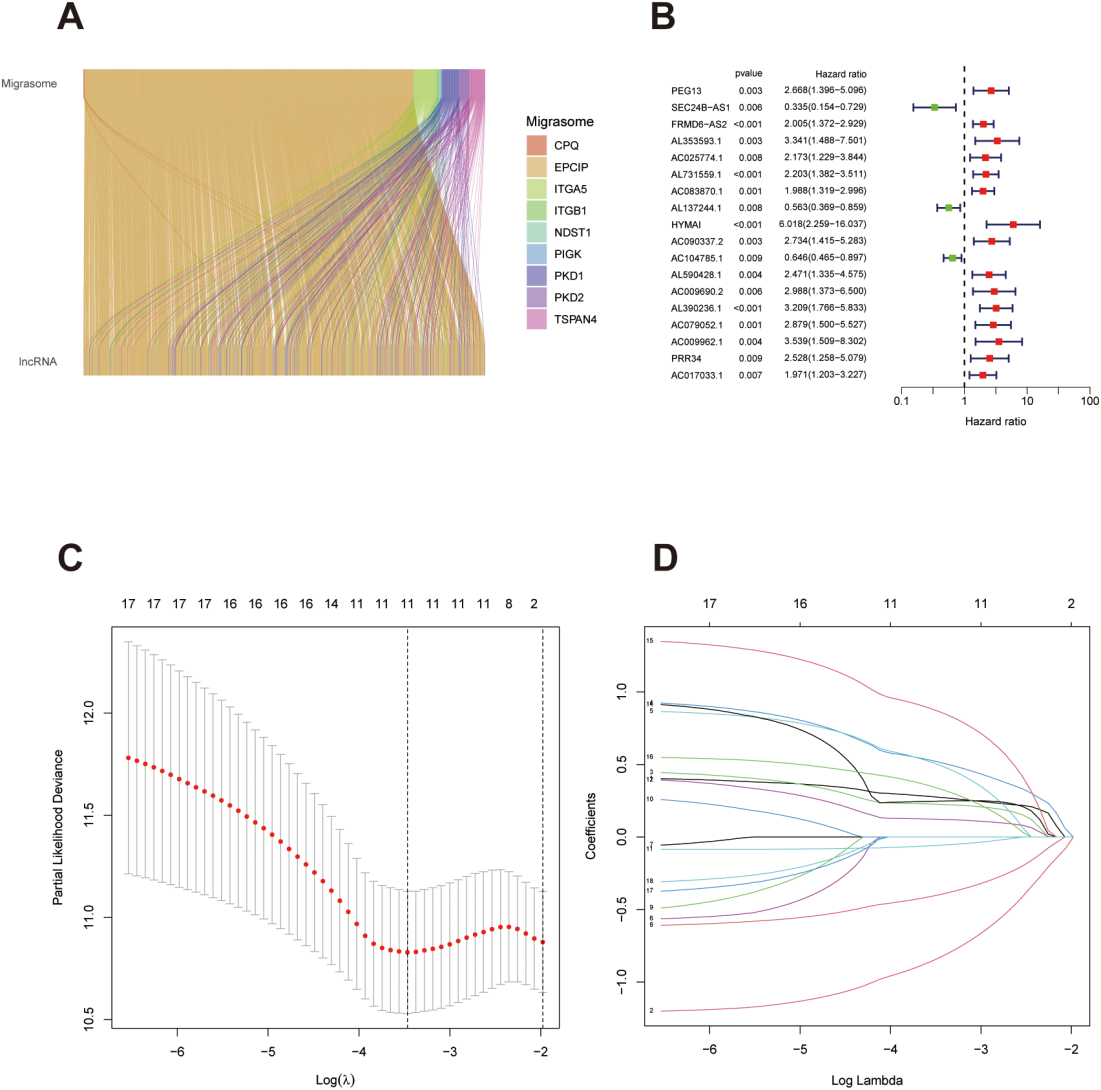
Screening of migrasome-associated lncRNAs and development of a prognostic model in bladder cancer. **(A)** Sankey diagram depicting the co-expression relationships between migrasome-related genes and corresponding lncRNAs in bladder cancer samples. **(B)** Forest plot summarizing the prognostic significance of selected lncRNAs in relation to overall survival (p < 0.001). HRs, Hazard ratios with 95% Cis, confidence intervals are presented; lncRNAs with HR > 1 are labeled in red (indicating increased risk), while those with HR < 1 are marked in green (indicating protective roles). **(C)** Coefficient profiles generated by LASSO regression illustrating the variation in model parameters across a range of log(λ) values, with the number of non-zero coefficients noted for each step. **(D)** Cross-validation curve used to identify the optimal penalty parameter λ, minimizing the mean cross-validated error for the final prognostic model.

Transcriptome data for BLCA were retrieved from the TCGA database, comprising 412 tumor samples and 19 adjacent normal samples. From these data, the expression levels of the selected migrasome-related genes were extracted to construct the gene expression matrix. Subsequently, long non-coding RNAs (lncRNAs) co-expressed with migrasome-related genes were screened using Pearson correlation analysis. LncRNAs showing a correlation coefficient |R| > 0.4 and a p-value < 0.001 were considered significantly associated (**Figure 1A**).

The full cohort was randomly split into a training cohort and a validation cohort. A comparison of clinical characteristics between the two groups confirmed the absence of significant differences (**Supplementary Table 2**), ensuring the comparability of downstream analyses. In the training cohort, univariate Cox regression was performed to identify survival-related lncRNAs (**Figure 1B**). These candidates were further refined through Lasso regression with cross-validation to construct a regularized risk model (**Figure 1C**). The lncRNAs retained from Lasso were subsequently incorporated into a multivariate Cox regression model to build the prognostic signature (**Figure 1D**). Stepwise model selection was applied to derive the final optimized signature.The formula for the risk score is as follows:


Risk score=−1.35130913390337∗SEC24B−AS1 + 1.14510410676361∗AL353593.1 + 0.816108062046749∗AC025774.1−0.49033863948232∗AL137244.1 + 1.29451040772368∗AC079052.1 + 0.8702290852544∗AC009962.1

After establishing the risk score model based on migrasome-associated lncRNAs in the training cohort, we proceeded to validate its prognostic utility in an independent validation cohort. For each patient in the training cohort, a risk score was computed using the established formula. Individuals were then stratified into high- or low-risk groups using the median risk score as the cutoff. The same median threshold derived from the training cohort was applied to classify patients in the validation cohort. To confirm the comparability of clinical characteristics between risk groups, we assessed variables such as age, gender, tumor grade, and stage using chi-square tests. In both cohorts, all comparisons yielded p-values greater than 0.05, suggesting no significant clinical imbalance between the high- and low-risk groups. These findings indicate that the stratification was not confounded by baseline heterogeneity and that the cohorts were suitable for downstream survival analyses.

**Figure 2:**
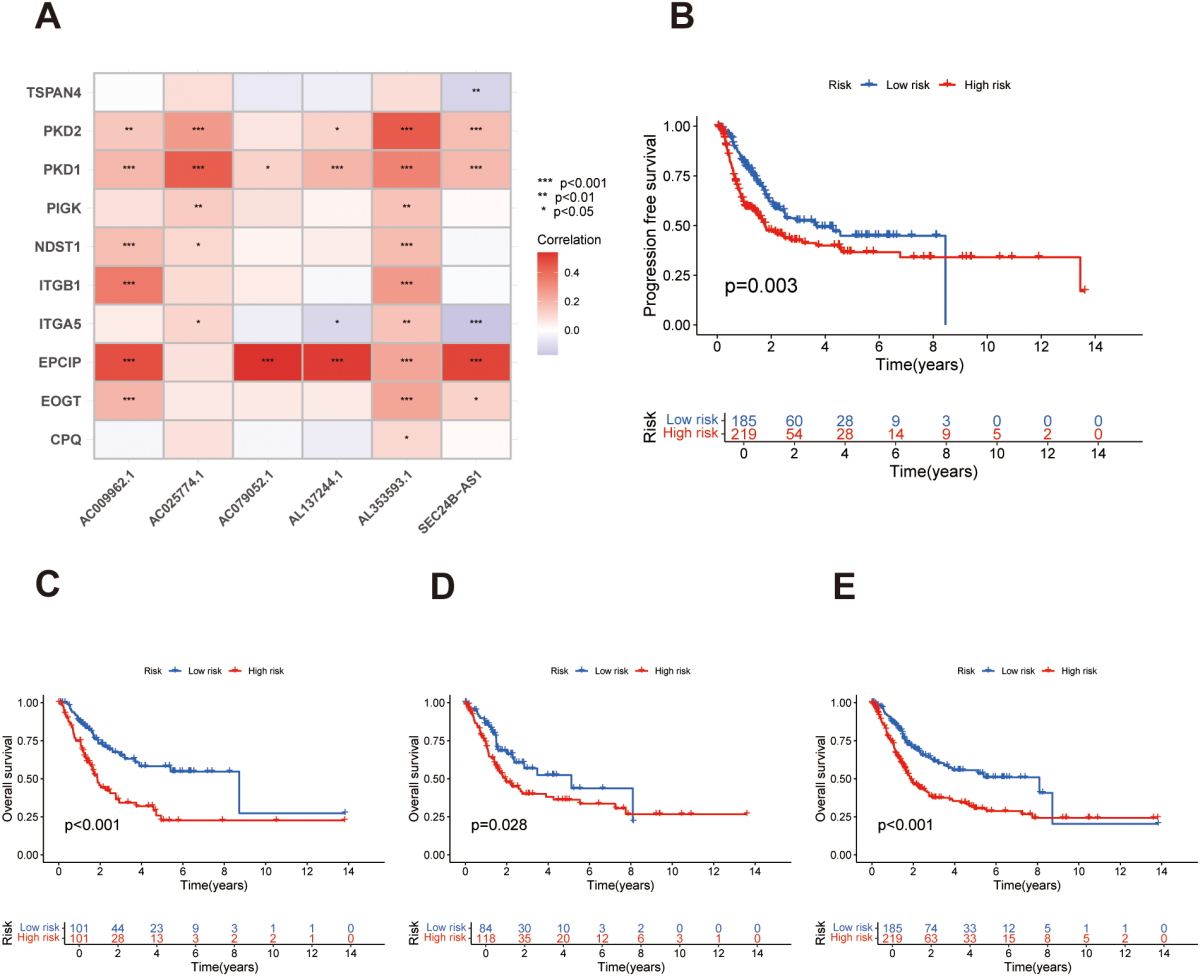
Prognostic impact and correlation features of the migrasome-related lncRNA risk model in bladder cancer. **(A)** Heatmap demonstrating Pearson correlation coefficients between migrasome-associated genes and the prognostic lncRNAs identified in the model. **(B)** PFS, Progression-free survival analysis comparing risk groups, with statistical significance evaluated via the log-rank test. **(C)** Kaplan–Meier survival analysis of OS, overall survival in the training cohort, showing distinct outcomes between low- and high-risk groups based on the lncRNA-based signature. **(D)** Kaplan–Meier survival analysis of OS, overall survival in the validation cohort, showing distinct outcomes between low- and high-risk groups based on the lncRNA-based signature. **(E)** Kaplan–Meier survival analysis of OS, overall survival in the entire cohort, showing distinct outcomes between low- and high-risk groups based on the lncRNA-based signature.*p < 0.05, **p < 0.01, and ***p < 0.001.

We first investigated the correlation between migrasome-related genes and the lncRNAs incorporated into the prognostic model. As shown in the correlation analysis, diverse degrees of association were observed across gene–lncRNA pairs, with most exhibiting moderate to strong co-expression patterns (**Figure 2A**). To further evaluate the prognostic relevance of the signature, we examined survival differences between the high- and low-risk groups. Kaplan–Meier analysis revealed that patients in the high-risk group had significantly worse progression-free survival (PFS) compared to those in the low-risk group (**Figure 2B**), suggesting a strong link between higher risk scores and faster disease progression. Consistent results were observed in the analysis of overall survival (OS). In the training cohort, the high-risk group displayed markedly shorter OS than the low-risk group (**Figure 2C**). This survival disadvantage remained evident in the validation cohort and was further confirmed in the entire BLCA cohort (**Figures 2D, E**), supporting the robustness and generalizability of the signature. Taken together, these findings highlight the strong prognostic power of the migrasome-associated lncRNA signature in predicting adverse clinical outcomes in bladder cancer.

**Figure 3:**
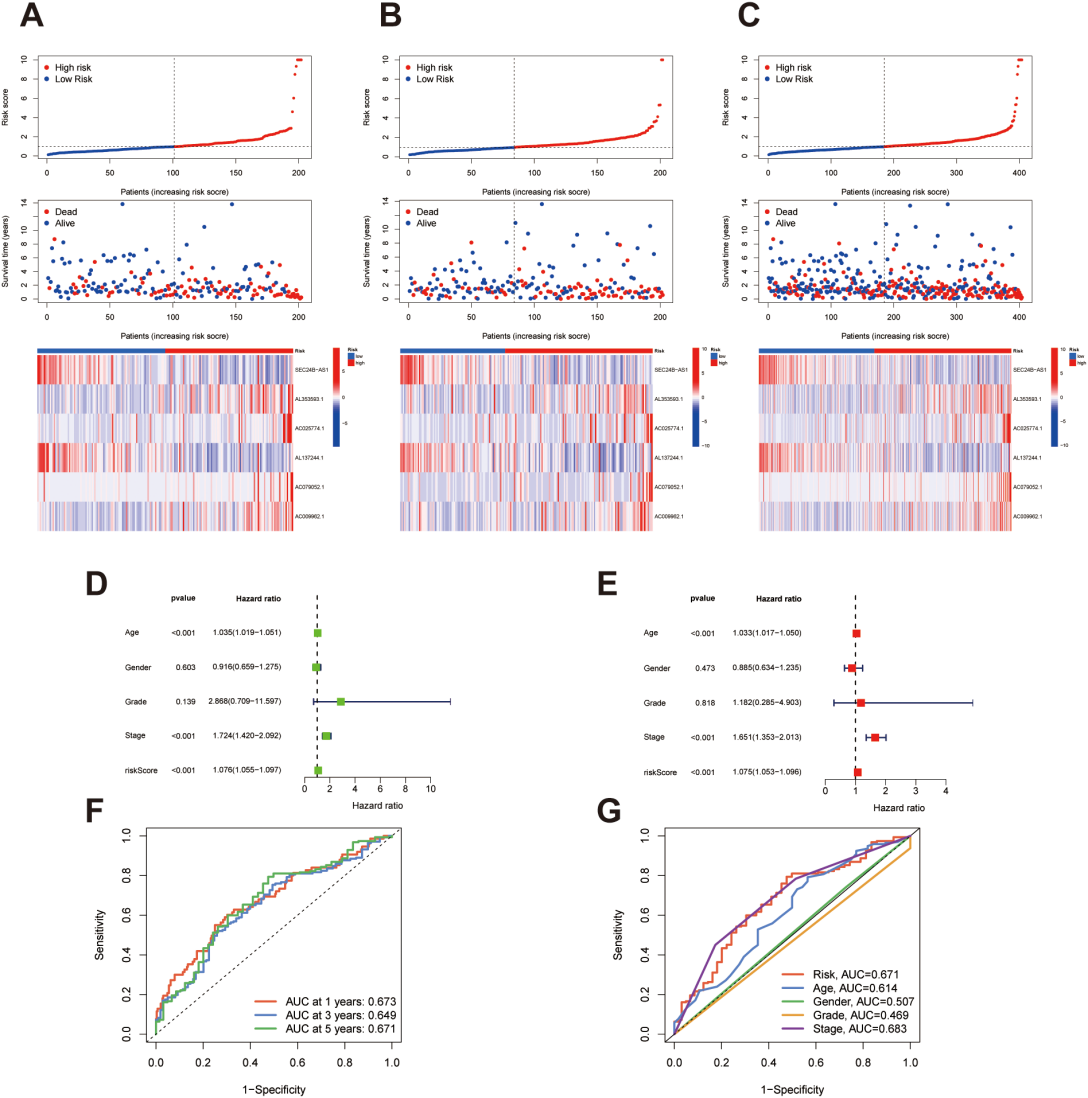
Evaluation of risk stratification and prognostic performance of the migrasome-associated lncRNA model in bladder cancer. **(A–C)** Distribution of risk scores, corresponding survival status, and expression heatmaps of the selected lncRNAs for the training, validation, and entire cohorts, respectively. Samples are ranked by ascending risk scores and categorized into high- and low-risk subgroups. **(D)** Results from univariate Cox regression showing HR, hazard ratios and statistical significance of clinical characteristics and the lncRNA-based model with respect to overall survival. **(E)** Multivariate Cox regression analysis confirming the independent prognostic relevance of the risk score after adjusting for conventional clinical parameters. **(F)** Time-dependent ROC analysis at 1, 3, and 5 years indicating the model’s predictive reliability over time. **(G)** Comparative ROC curves for 5-year OS prediction illustrating that the migrasome-related lncRNA signature outperforms traditional clinicopathological features.

Using the risk scores derived from the migrasome-related lncRNA signature, all patients were ranked from lowest to highest and grouped into high- and low-risk categories based on the median score. Visual inspection of survival status distributions revealed a clear increase in the number of deceased patients in the high-risk group across all datasets. In parallel, heatmap analyses revealed distinct expression patterns of the signature lncRNAs between the two risk groups, which were consistently observed in the training, validation, and entire cohorts (**Figures 3A–C**). These observations reinforce the signature’s ability to distinguish patients with poor prognosis. To assess the prognostic relevance of the signature in the context of clinical variables, we performed Cox proportional hazards regression analysis. Univariate analysis identified several factors—age, tumor grade, clinical stage, and the lncRNA-based risk score—as significantly associated with overall survival (**Figure 3D**). These variables were then included in a multivariate Cox regression model to examine their independent effects. The risk score remained a significant predictor of survival after adjusting for confounders, suggesting its role as an independent prognostic factor distinct from conventional clinical features (**Figure 3E**). ROC curve analysis was then conducted to evaluate the signature’s predictive performance. The risk model showed favorable accuracy for predicting 1-, 3-, and 5-year survival, with area under the curve (AUC) values demonstrating strong prognostic utility (**Figure 3F**). Furthermore, comparative analysis revealed that the risk score outperformed other clinical variables, including age, sex, grade, and stage, in predicting patient outcomes, confirming the superior predictive capability of the migrasome-associated lncRNA signature (**Figure 3G**).

**Figure 4:**
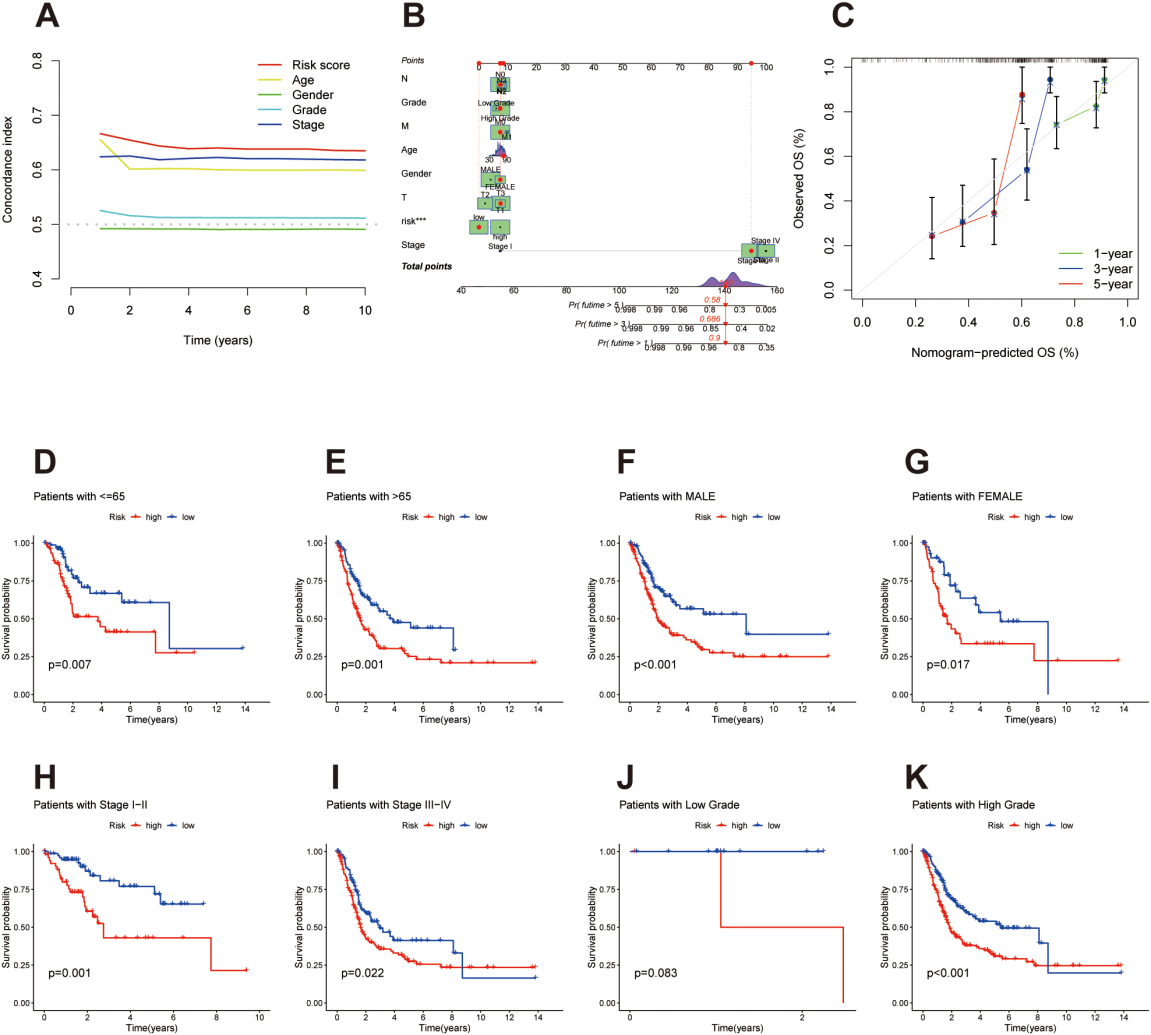
Prognostic reliability and subgroup validation of the migrasome-related lncRNA signature in bladder cancer. **(A)** C-Index, Concordance index comparison between the lncRNA-based risk score and conventional clinical features (age, gender, grade, stage), with the risk model showing the highest prognostic accuracy (C-index = 0.80). **(B)** Nomogram incorporating the risk score and clinical variables to predict 1-, 3-, and 5-year overall survival probabilities. **(C)** Calibration curves assessing the agreement between predicted and actual survival outcomes at 1, 3, and 5 years, confirming the nomogram’s predictive accuracy. **(D, E)** Kaplan-Meier survival curves stratified by age groups (≤65 and >65 years), with high-risk patients in both subgroups showing significantly poorer survival. **(F, G)** Kaplan-Meier survival curves based on gender, indicating consistent prognostic discrimination between high- and low-risk groups in both male and female patients. **(H, I)** Kaplan-Meier survival curves in patients with early (stage I–II) and advanced disease (stage III–IV), confirming prognostic value across clinical stages. **(J, K)** Kaplan-Meier survival curves by tumor grade: although low-grade samples were insufficient for effective comparison, a significant survival difference was observed in high-grade patients, favoring the low-risk group.

To evaluate the predictive performance of the migrasome-related lncRNA signature, we calculated the concordance index (C-index) for the risk score and compared it with conventional clinical factors. The risk score achieved the highest C-index value, reaching 0.80, outperforming age, gender, grade, and stage in survival prediction (**Figure 4A**). A nomogram integrating the risk score with key clinical variables was constructed to facilitate individualized survival prediction (**Figure 4B**). Calibration plots demonstrated strong agreement between predicted and observed overall survival (OS) probabilities at 1, 3, and 5 years, indicating reliable predictive performance of the nomogram (**Figure 4C**). To assess the consistency of the prognostic value of the migrasome-associated lncRNA signature across different clinical conditions, we conducted subgroup survival analyses. Among patients aged ≤65 and >65 years, the risk signature remained an effective prognostic indicator. In both age groups, individuals categorized as high-risk experienced significantly worse overall survival compared to their low-risk counterparts (**Figures 4D, E**). A similar pattern was observed in sex-stratified analysis: male and female patients with higher risk scores consistently exhibited poorer survival outcomes than those with lower scores (**Figures 4F, G**). Analysis across clinical stages revealed that the signature maintained prognostic relevance in both early-stage (I–II) and advanced-stage (III–IV) patients, with the high-risk group demonstrating a clear survival disadvantage in each case (**Figures 4H, I**). In the low-grade subgroup, effective analysis could not be performed due to the limited number of clinical samples. However, in the high-grade subgroup, patients in the high-risk group exhibited significantly worse prognoses compared to those in the low-risk group (**Figures 4J, K**).

**Figure 5:**
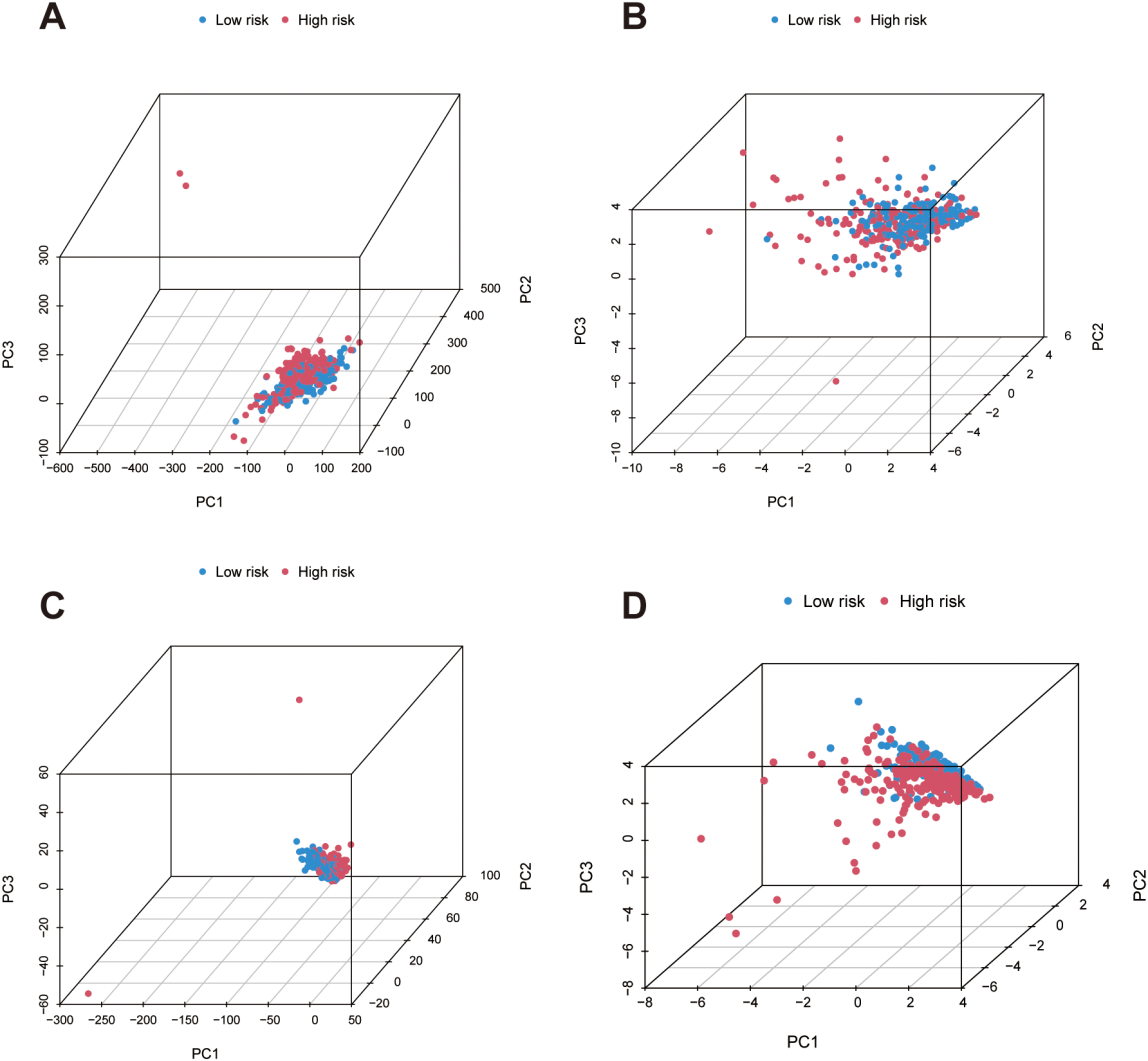
Principal component analysis reveals distinct risk group distribution based on gene expression patterns in bladder cancer. **(A)** PCA based on the complete transcriptome shows overlapping distributions between high- and low-risk groups, suggesting limited discriminatory capacity. **(B)** PCA plots derived from migrasome-related gene sets shows enhanced separation of the two risk categories, indicating better discriminatory capacity. **(C)** PCA plots derived from migrasome-related co-expressed lncRNAs shows enhanced separation of the two risk categories, indicating better discriminatory capacity. **(D)** When applying PCA using the lncRNAs included in the prognostic signature, a clear distinction between high- and low-risk groups is observed, emphasizing the robust stratification power of the established signature.

To explore the distribution patterns of patients with different risk levels, principal component analysis (PCA) was conducted across four datasets in the context of bladder cancer. When using the global transcriptomic data, high- and low-risk patients did not display clear segregation, indicating that overall gene expression was not sufficient to distinguish between the two groups (**Figure 5A**). However, when PCA was based on genes associated with migrasomes or their related lncRNAs, a more evident clustering pattern emerged, implying their relevance in risk categorization (**Figures 5B, C**). Notably, when we applied PCA using the lncRNAs included in our prognostic signature, the separation between high- and low-risk groups became more pronounced, underscoring the robustness and discriminative power of the selected lncRNAs in stratifying patient outcomes (**Figure 5D**).

### 3.2 Functional enrichment analysis reveals distinct biological characteristics between risk groups in bladder cancer

**Figure 6:**
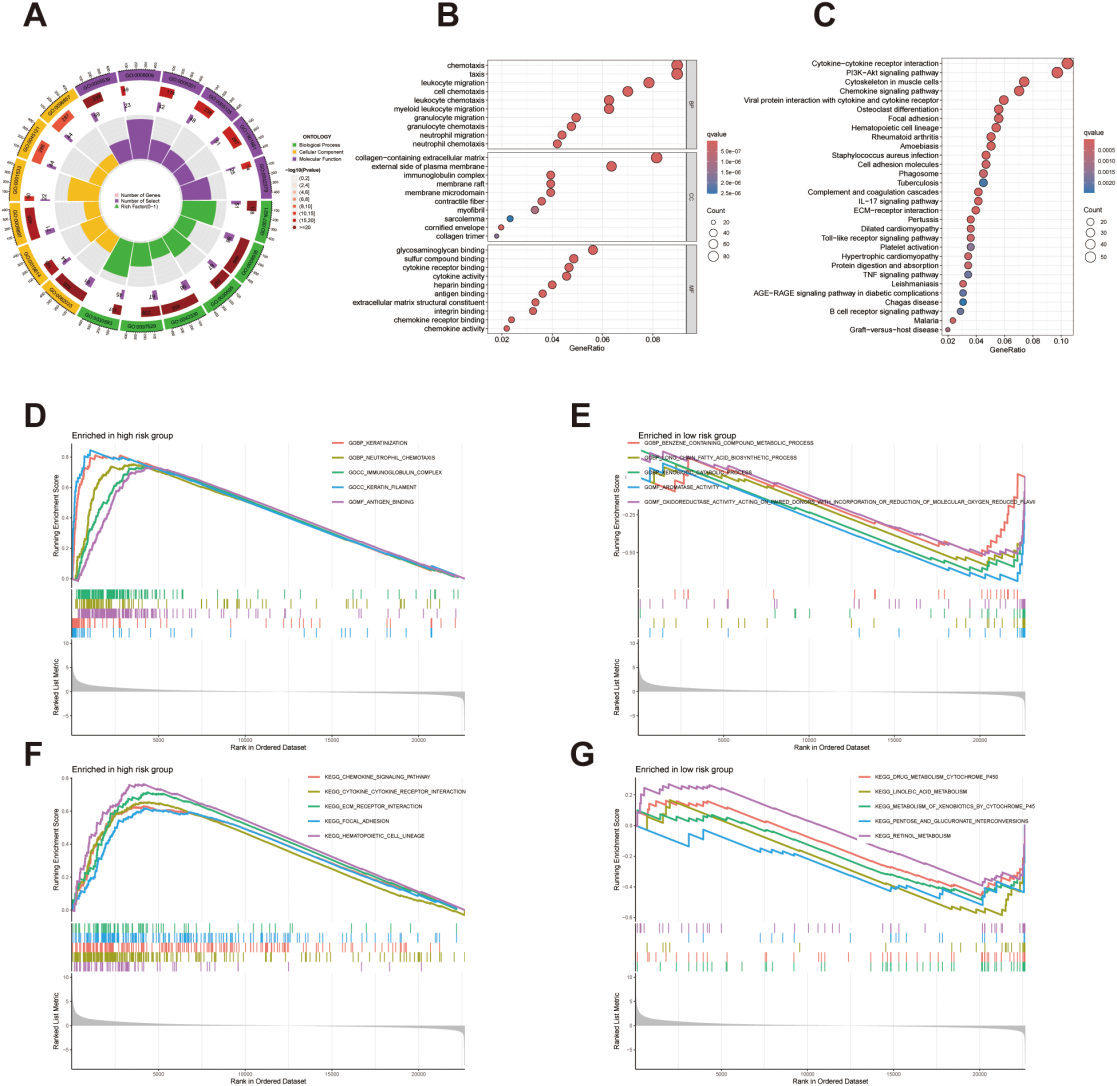
Functional enrichment analysis of differentially expressed genes between high- and low-risk groups. **(A)** Circular plot showing the distribution and classification of DEGs across major functional categories. **(B)** GO enrichment analysis of DEGs across biological process, cellular component, and molecular function categories. **(C)** KEGG pathway enrichment analysis identifying immune- and stroma-related pathways enriched in high-risk patients. **(D, E)** GSEA plots illustrate the five most significantly enriched functions distinguishing the high-risk and low-risk patient cohorts. **(F, G)** GSEA plots illustrate the five most significantly enriched pathways distinguishing the high-risk and low-risk patient cohorts.

To explore the biological implications of gene expression differences between high- and low-risk bladder cancer patients, we conducted comprehensive enrichment analyses. A circular diagram was generated to illustrate the overall distribution and proportional contribution of differentially expressed genes (DEGs) across major functional categories (**Figure 6A**). Gene Ontology (GO) enrichment analysis revealed that biological processes were predominantly related to immune cell migration and chemotaxis, including neutrophil chemotaxis, leukocyte migration, and granulocyte chemotaxis. In the cellular component category, DEGs were enriched in collagen-containing extracellular matrix, immunoglobulin complex, and external side if plasma membrane. Molecular function analysis highlighted significant enrichment in chemokine activity, integrin binding, and glycosaminoglycan binding, suggesting active involvement in extracellular matrix remodeling and immune signaling (**Figure 6B**). Further pathway enrichment analysis using the Kyoto Encyclopedia of Genes and Genomes (KEGG) identified several immune-related and stromal pathways. Key enriched pathways included cytokine–cytokine receptor interaction, PI3K-Akt signaling pathway, ECM–receptor interaction, chemokine signaling pathway, Toll-like receptor signaling, and complement and coagulation cascades, all of which are functionally associated with tumor–immune crosstalk and microenvironmental modulation (**Figure 6C**). To distinguish functional trends between risk groups, Gene Set Enrichment Analysis (GSEA) was conducted. The high-risk subgroup exhibited upregulation of immune-related functions, including neutrophil chemotaxis, immunoglobulin complex formation, and antigen binding, alongside keratinization-associated processes (**Figure 6D**). Conversely, the low-risk group demonstrated enrichment in metabolic pathways such as benzene-containing compound metabolism, long-chain fatty acid biosynthesis, and xenobiotic catabolic processes, suggesting metabolic reprogramming underpins prognostic stratification (**Figure 6E**). Pathway-level GSEA revealed that high-risk patients showed enhanced activity in immune system pathways and extracellular matrix interactions, including chemokine signaling pathway, cytokine-cytokine receptor interaction, hematopoietic cell lineage, and focal adhesion (**Figure 6F**). In contrast, pathways related to metabolic processes—such as drug metabolism cytochrome p450, linoleic acid metabolism, metabolism of xenobiotics by cytochrome p450, and retinol metabolism—were predominantly enriched in the low-risk cohort (**Figure 6G**). These findings suggest that the lncRNA signature may reflect distinct immunological profiles linked to cytokine signaling and hematopoietic regulation in high-risk patients, while low-risk patients exhibit metabolic adaptations involving lipid and xenobiotic metabolism, highlighting potential mechanisms underlying tumor behavior in bladder cancer.

### 3.3 Immune landscape and tumor microenvironment characterization

**Figure 7:**
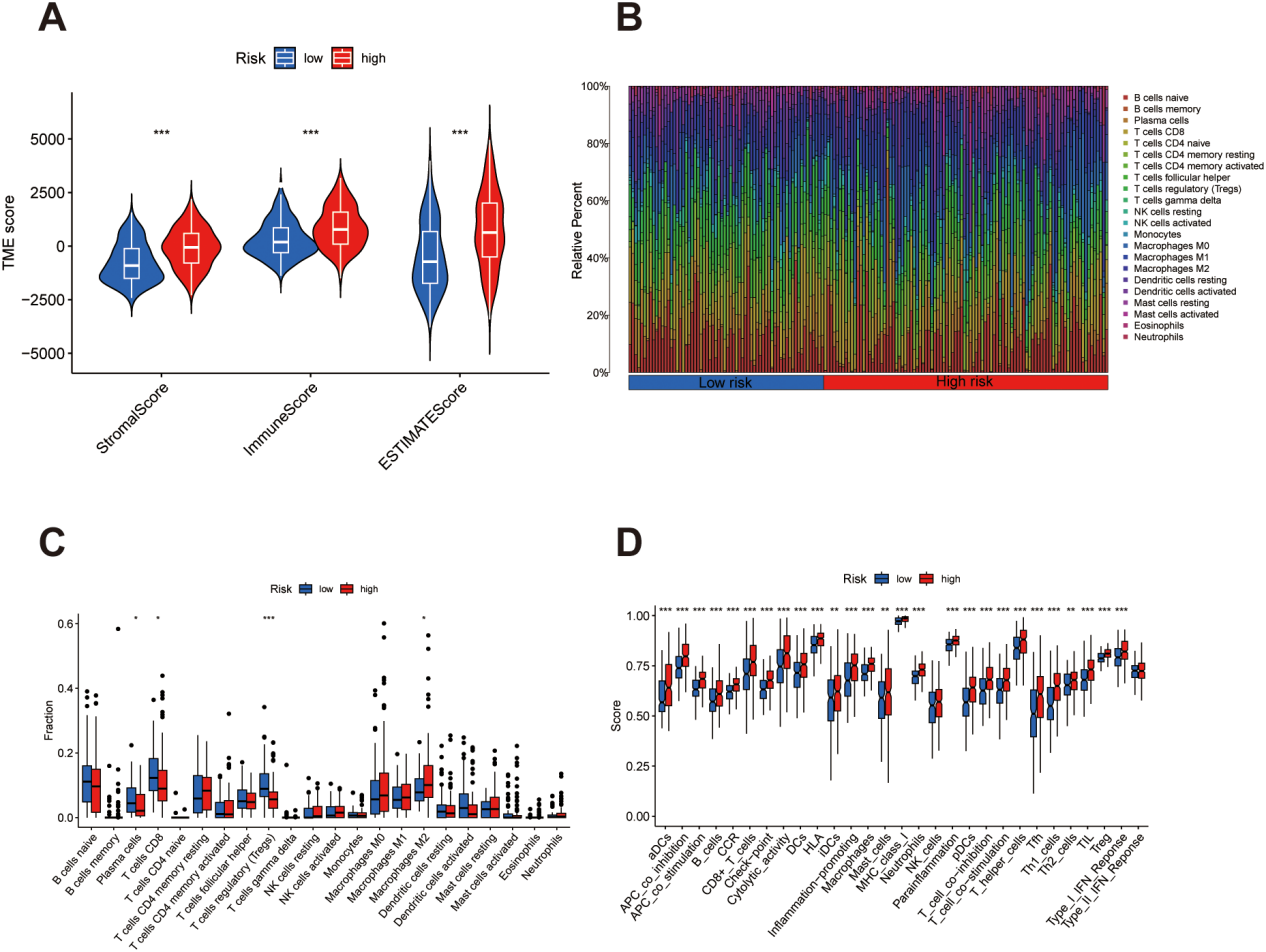
Divergent immune microenvironment landscapes in bladder cancer risk subgroups. **(A)** Violin plots illustrating differences in StromalScore, ImmuneScore, and ESTIMATEScore, with elevated levels predominantly observed in the high-risk cohort. **(B)** Bar chart displaying the proportional distribution of immune cell populations, revealing distinct immune infiltration profiles between the two groups. **(C)** Box plots depicting statistically significant variation in specific immune cell subsets, highlighting preferential enrichment patterns in each risk category. **(D)** Box plots comparing functional immune signatures, indicating enhanced immune activation or suppression mechanisms in the high-risk subgroup relative to low-risk patients. *p < 0.05, **p < 0.01, and ***p < 0.001.

To evaluate the tumor microenvironment (TME) landscape in relation to the risk signature, we compared stromal and immune components between high- and low-risk patients. Violin plot analysis revealed that StromalScore, ImmuneScore, and the combined ESTIMATEScore were all significantly elevated in the high-risk group (**Figure 7A**), suggesting a denser infiltration of stromal and immune cells in this subgroup. Next, we examined the distribution of specific immune cell populations across risk groups. As shown in the bar plot (**Figure 7B**), patients in the high-risk group displayed higher proportions of M2 macrophages, whereas the low-risk group was enriched in Plasma cells, CD8+ T cells and T cells regulatory (Tregs), reflecting divergent immune compositions. These trends were further substantiated by box plot analysis (**Figure 7C**), which highlighted statistically significant differences in several immune cell subsets between the two groups. Functional immune profiling using ssGSEA revealed differential enrichment of immune-related activities. The high-risk group exhibited elevated scores in functions such as inflammation-promoting and check points (**Figure 7D**), indicating a heightened but potentially dysregulated immune activation state. These results suggest that the prognostic signature is associated with distinct immunological characteristics within the tumor microenvironment of bladder cancer.

### 3.4 Genomic alterations and tumor mutation burden analysis

**Figure 8:**
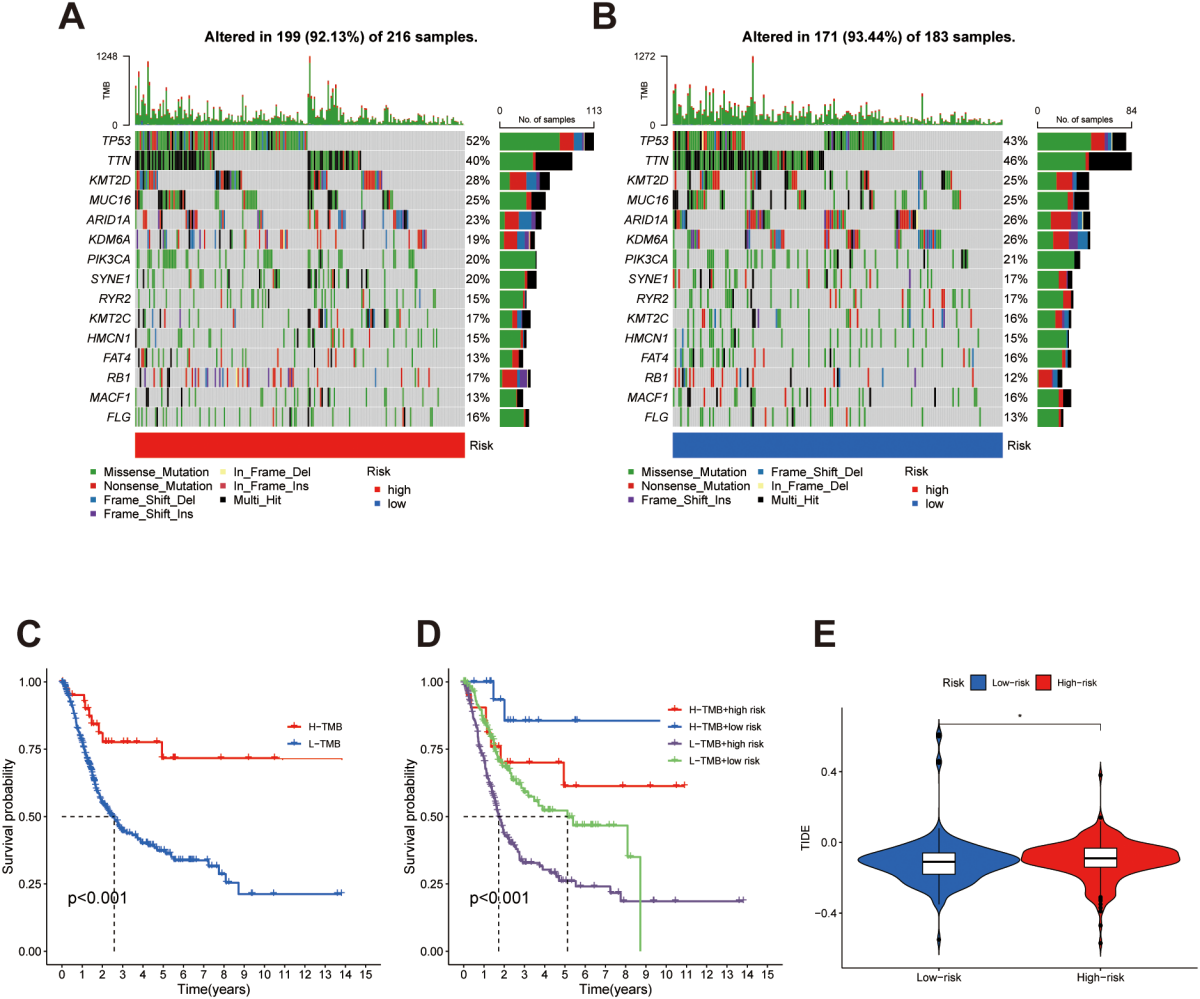
Tumor mutation burden and immune escape profiles in bladder cancer risk subgroups. **(A, B)** TMB distributions in the high- and low-risk groups, respectively, reveal a greater mutational load in low-risk patients. **(C)** Kaplan–Meier survival analysis stratified by TMB status indicates worse prognosis in patients with low TMB. **(D)** Integrated assessment of TMB and risk score stratification shows that the L-TMB + high-risk group exhibits the poorest survival, whereas the H-TMB + low-risk group has the most favorable outcome. **(E)** TIDE score analysis highlights a higher immune evasion tendency in the high-risk subgroup. *p < 0.05.

To assess the genomic differences between risk groups, we analyzed the somatic mutation profiles of the 15 most frequently altered genes in bladder cancer. Mutation landscape plots revealed that genes such as TP53 and TTN exhibited high mutation frequencies in both high- and low-risk groups (**Figures 8A, B**), though the mutation load appeared more pronounced in the low-risk subgroup.

We next explored the prognostic relevance of TMB alone. Kaplan-Meier survival analysis showed that individuals with low TMB had shorter overall survival than those with high TMB (**Figure 8C**), suggesting that less mutational events might contribute to more aggressive disease behavior. Furthermore, stratifying patients by both TMB status and risk score identified distinct survival patterns: the high-risk + low-TMB group exhibited the worst survival outcomes, while patients classified as low-risk + high-TMB demonstrated the most favorable prognosis (**Figure 8D**). These findings highlight the additive prognostic value of integrating genomic instability with transcriptomic risk. To evaluate immune evasion potential, we applied the TIDE algorithm. Results showed that TIDE scores were significantly higher in the high-risk group (**Figure 8E**), suggesting a greater likelihood of immune escape and potentially reduced responsiveness to immune checkpoint blockade in this subgroup.

### 3.5 Differential drug sensitivity patterns between risk groups in bladder cancer

**Figure 9:**
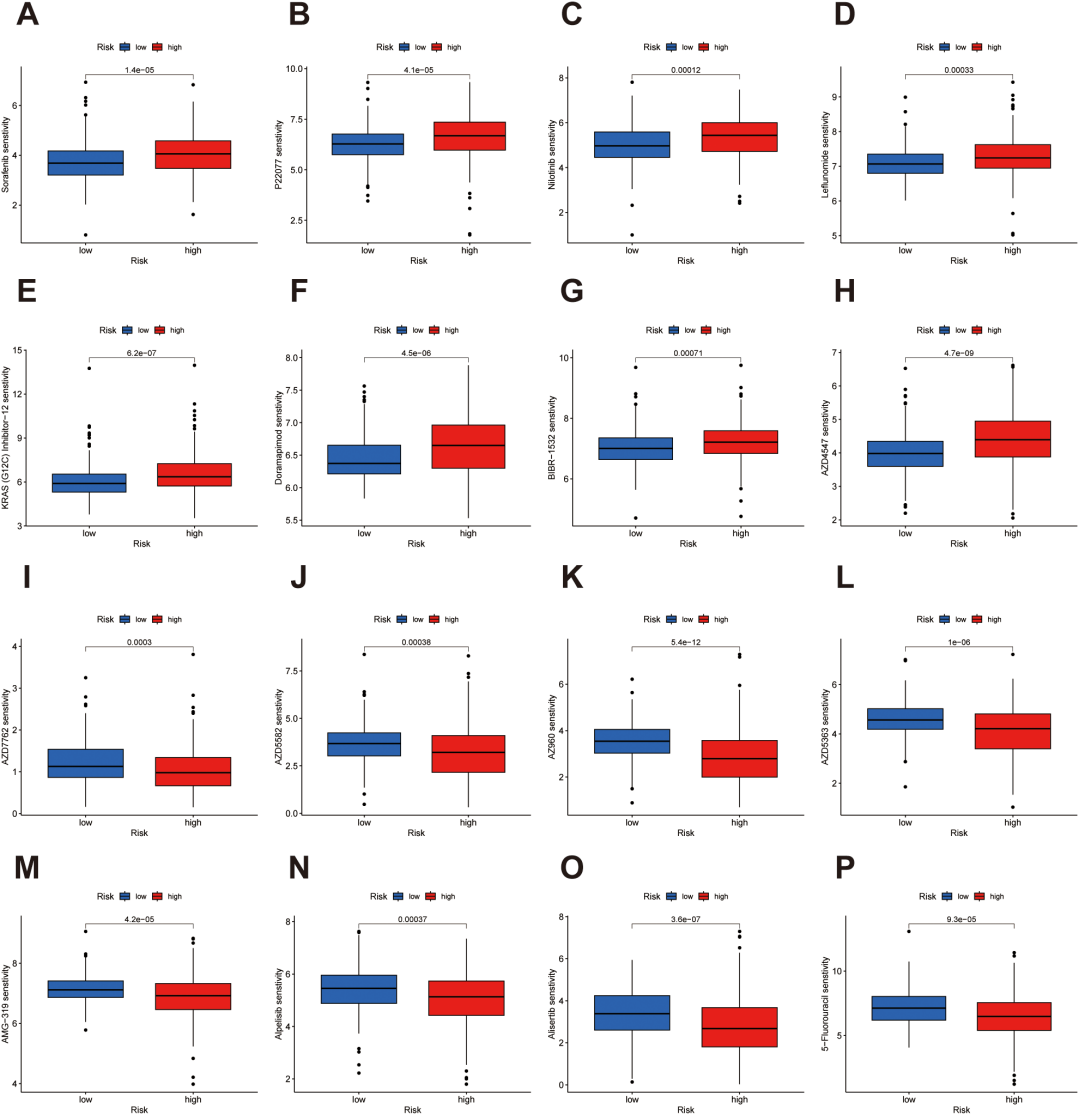
Drug sensitivity analysis reveals divergent therapeutic vulnerabilities between risk groups in bladder cancer. **(A–H)** The low-risk subgroup demonstrated increased predicted sensitivity to agents including Sorafenib, P22077, Nilotinib, Leflunomide, KRAS(G12C) Inhibitor, Doramapimod, BIBR-1532, and AZD4547. **(I–P)** Conversely, the high-risk group showed heightened susceptibility to AZD7762, AZD5582, AZ960, AZD5363, AMG-319, Alpelisib, Alisertib, and 5-Fluorouracil.

To explore potential treatment avenues based on our prognostic classification, we conducted a drug sensitivity analysis comparing high- and low-risk groups. The analysis identified multiple compounds with differential predicted efficacy across the two cohorts. Patients classified into the low-risk group exhibited greater sensitivity to several agents, including Sorafenib, P22077, Nilotinib, Leflunomide, KRAS(G12C) Inhibitor, Doramapimod, BIBR-1532, and AZD4547 (**Figures 9A–H**). These compounds primarily target cellular energy metabolism, intracellular signaling pathways and epigenetic regulation, suggesting that low-risk patients may benefit more from therapies directed at metabolic and proliferative signaling mechanisms. On the other hand, individuals in the high-risk subgroup showed increased responsiveness to distinct compounds such as AZD7762, AZD5582, AZ960, AZD5363, AMG-319, Alpelisib, Alisertib, and 5-Fluorouracil (**Figures 9I–P**). These agents predominantly act on apoptotic signaling, cell cycle regulation, and immune modulation pathways, indicating that high-risk patients may be more amenable to treatment strategies targeting survival signaling and the tumor microenvironment. A broader spectrum of drug sensitivity results is available in **Supplementary Figures 1**, **S2**, offering a more comprehensive view of pharmacologic vulnerabilities across the stratified groups. Collectively, these findings underscore the potential of incorporating risk-based drug prediction models into personalized treatment strategies for bladder cancer patients.

### 3.6 Single-cell transcriptomic profiling uncovers cellular diversity and lncRNA-signature–related microenvironmental characteristics in BLCA

**Figure 10:**
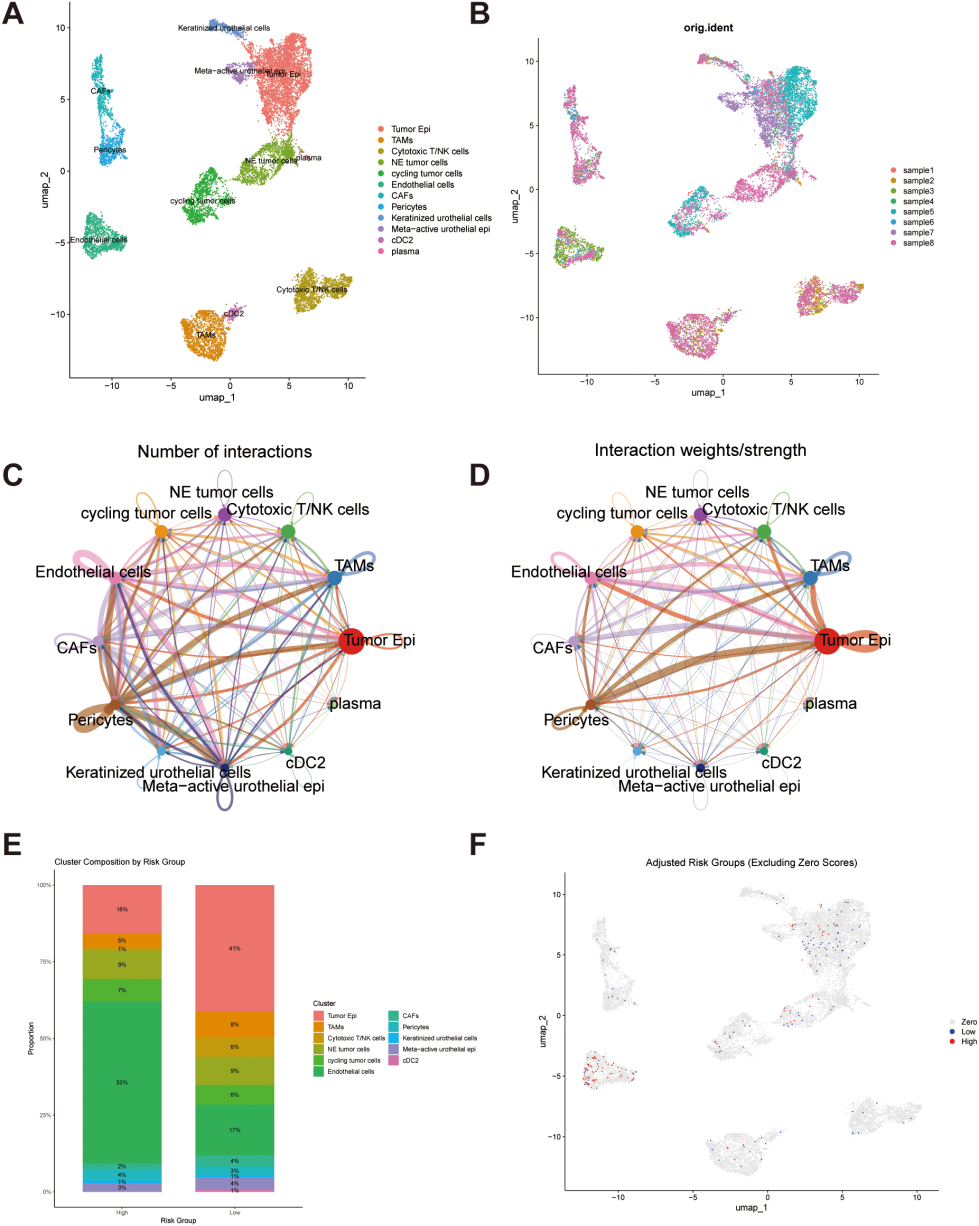
Single-cell transcriptomic landscape and risk group distribution in bladder cancer. **(A)** UMAP visualization showing the distinct clustering of major cell populations identified across all BLCA samples, including urothelial tumor epithelial cells, TAMs, tumor-associated macrophages, cytotoxic T/NK cells, neuroendocrine-like tumor cells, cycling tumor cells, endothelial cells, CAFs, cancer-associated fibroblasts, pericytes, keratinized urothelial cells, metabolically active urothelial epithelial cells, conventional dendritic cells type 2 (cDC2), and plasma cells. **(B)** UMAP plot illustrating the distribution of cells by individual sample after batch correction. **(C)** Cell–cell interaction network displaying the predicted number of communications among various cell types. **(D)** Network visualization weighted by interaction strength, highlighting key cellular hubs within the BLCA microenvironment. **(E)** Stacked bar plot showing the relative proportions of different cell types within the high- and low-risk groups. **(F)** UMAP plot representing the spatial distribution of single-cell risk scores derived from the prognostic signature.

To further evaluate the ability of our prognostic signature to characterize cellular diversity and intercellular communication patterns within the bladder cancer (BLCA) tumor microenvironment, publicly available single-cell RNA sequencing (scRNA-seq) datasets were analyzed. After performing standard preprocessing procedures—including quality filtering, normalization, and dimensionality reduction—Uniform Manifold Approximation and Projection (UMAP) visualization delineated distinct cellular clusters representing major cell populations. These included urothelial tumor epithelial cells, tumor-associated macrophages (TAMs), cytotoxic T/NK cells, neuroendocrine-like tumor cells, cycling tumor cells, endothelial cells, cancer-associated fibroblasts (CAFs), pericytes, keratinized urothelial cells, metabolically active urothelial epithelial cells, conventional type 2 dendritic cells (cDC2), and plasma cells (**Figure 10A**). Cells derived from different patients were evenly distributed across these clusters, suggesting minimal batch-dependent variability after correction (**Figure 10B**). Cell–cell communication analysis revealed that tumor epithelial cells, pericytes, and cytotoxic T/NK cells occupied central positions within the interaction network, establishing the most extensive intercellular connections (**Figure 10C**). Furthermore, these populations exhibited the strongest communication intensities, indicating that they may serve as critical regulators orchestrating the cellular interactions that shape the BLCA tumor microenvironment (**Figure 10D**). To explore how the prognostic signature relates to the tumor microenvironment at the single-cell level, each cell was assigned a risk score based on the expression of the signature genes. Cells with zero scores were removed to reduce potential bias from low sequencing coverage. The remaining cells were then divided into high- and low-risk categories using the median risk score as the threshold. Analysis of cellular composition revealed notable differences between these two groups, with endothelial cells predominantly represented within the high-risk fraction (**Figures 10E, F**). The observed differences in immune and stromal characteristics between high- and low-risk cells imply that variations in the tumor microenvironment are closely related to the divergent clinical prognoses linked to the risk signature.

**Figure 11:**
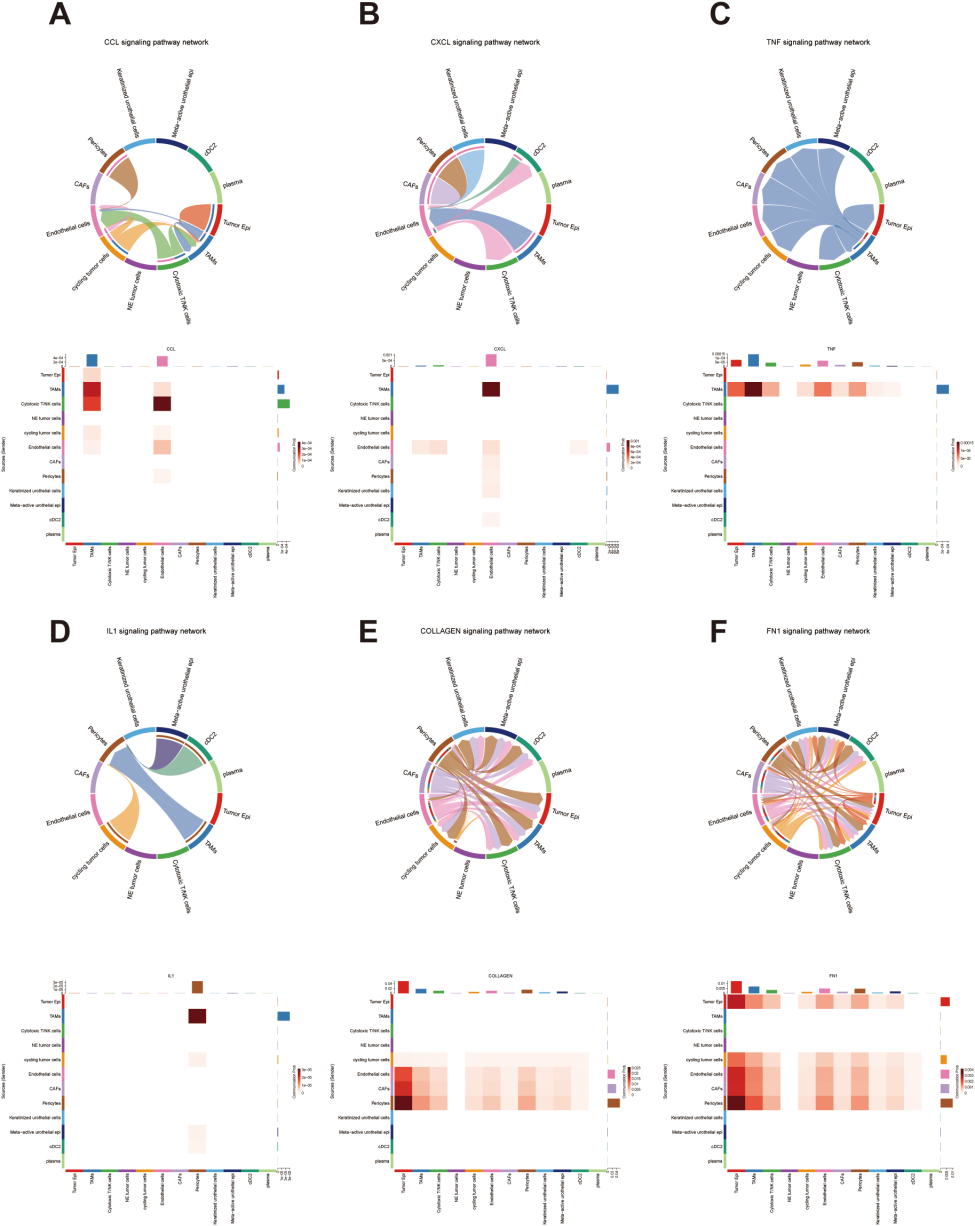
Intercellular communication networks associated with enriched signaling pathways in bladder cancer. **(A)** Predicted cell–cell communication network of the CCL signaling pathway. **(B)** Predicted cell–cell communication network of the CXCL signaling pathway. **(C)** Predicted cell–cell communication network of the TNF signaling pathway. **(D)** Predicted cell–cell communication network of the IL1 signaling pathway. **(E)** Predicted cell–cell communication network of the COLLAGEN signaling pathway. **(F)** Predicted cell–cell communication network of the FN1 signaling pathway.

Building upon the previous functional enrichment analysis (**Figure 6**), we next investigated how these enriched signaling pathways were mediated through intercellular communication at the single-cell level. Given the prominent enrichment of chemokine-associated pathways such as *KEGG_CHEMOKINE_SIGNALING_PATHWAY* in the high-risk group, we focused on core chemokine signaling modules, including the CCL and CXCL families. The analysis revealed that tumor-associated macrophages (TAMs) and cytotoxic T/NK cells served as the major signal senders, whereas TAMs and endothelial cells acted as the primary receivers (**Figures 11A, B**). TAMs and cytotoxic T/NK cells jointly functioned as dominant sources of chemokine signals, potentially linked to immune cell recruitment and polarization signatures. In contrast, TAMs and endothelial cells, as the principal receivers, may amplify chemokine-mediated signaling, which correlates with phenotypes of immunosuppression and angiogenesis within the tumor microenvironment. Furthermore, enrichment of the *KEGG_CYTOKINE_CYTOKINE_RECEPTOR_INTERACTION* pathway prompted further investigation of cytokines such as TNF and IL1 involved in this signaling axis. In the TNF-related signaling network, TAMs emerged as the predominant senders, transmitting signals to tumor epithelial cells, TAMs themselves, and cytotoxic T/NK cells (**Figure 11C**). Similarly, in IL1-associated communication, TAMs remained the main signal source, while pericytes represented the major signal receivers (**Figure 11D**). These findings suggest that TAMs, through secretion of key cytokines such as TNF and IL1, engage in extensive cross-talk with tumor epithelial cells, T/NK cells, and vascular-associated cells, collectively contributing to immune suppression, vascular remodeling, and tumor progression. We also examined adhesion-related signaling pathways based on the enrichment of *KEGG_FOCAL_ADHESION* in the high-risk group, with a particular focus on collagen and fibronectin (FN1)-mediated communication (**Figure 11E**). In the collagen signaling network, endothelial cells, CAFs, and pericytes were identified as the primary signal senders, whereas tumor epithelial cells and TAMs served as the major receivers. Similarly, in FN1-related interactions, tumor epithelial cells, cycling tumor cells, endothelial cells, CAFs, and pericytes acted as major signal sources, with tumor epithelial cells and TAMs again functioning as key receivers (**Figure 11F**). Collectively, these results demonstrate that in bladder cancer, focal adhesion signaling networks centered on collagen and fibronectin are co-driven by stromal cells (CAFs, endothelial cells, and pericytes) and tumor cells. Such extensive adhesion-mediated signaling indicates that the malignant phenotype of high-risk tumors arises not only from intrinsic tumor cell properties but also from the dynamic, bidirectional interactions between tumor cells and the stroma, accompanied by extracellular matrix (ECM) remodeling. This reciprocal communication network is highly associated with features of tumor progression, immune evasion, and enhanced metastatic potential.

**Figure 12:**
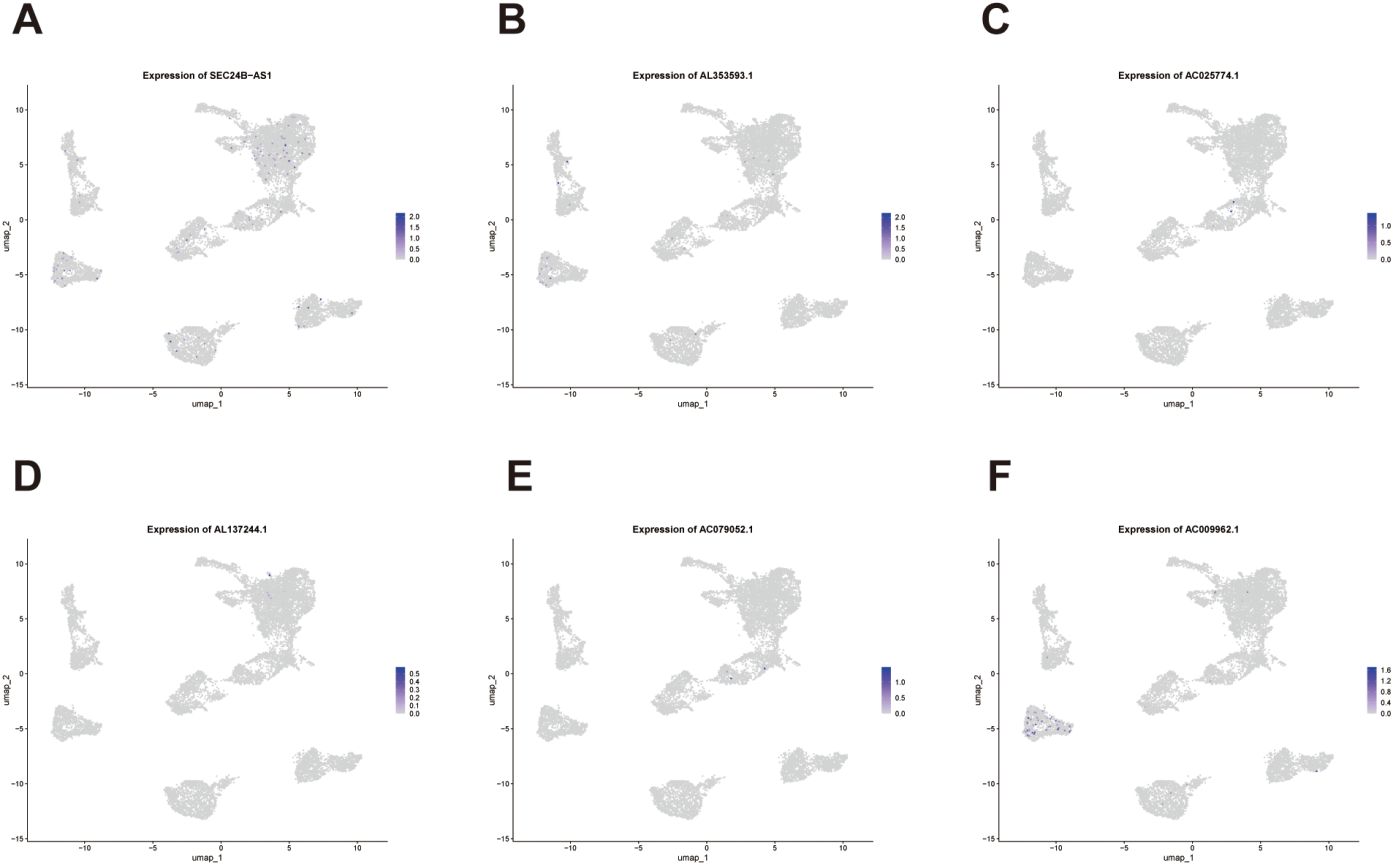
Single-cell expression landscape of prognostic lncRNAs across major cell populations in bladder cancer. **(A–F)** FeaturePlot visualization showing the expression patterns of the six lncRNAs comprising the prognostic signature (SEC24B-AS1, AL353593.1, AC025774.1, AL137244.1, AC079052.1, and AC009962.1) across distinct cellular clusters in the integrated single-cell dataset.

To further examine the cell type–specific expression patterns of the six lncRNAs comprising our prognostic signature, we performed *FeaturePlot* visualization using the integrated single-cell dataset. The results revealed heterogeneous expression distributions of these genes across distinct cellular clusters (**Figures 12A–F**). Notably, SEC24B-AS1 displayed a relatively broad expression profile, with higher expression levels observed in both tumor epithelial and endothelial cells. In contrast, AC009962.1 expression appeared to be more confined to endothelial cells. These findings suggest that SEC24B-AS1 may function as a multifunctional lncRNA associated with cross–cell type networks, potentially acting as a critical molecular link between tumor cells and the microenvironment.

This observation prompted us to pursue further experimental validation centered on SEC24B-AS1 to strengthen the biological relevance and robustness of our prognostic signature.

### 3.7 Experimental validation of SEC24B-AS1 as a key component of the prognostic signature

**Figure 13:**
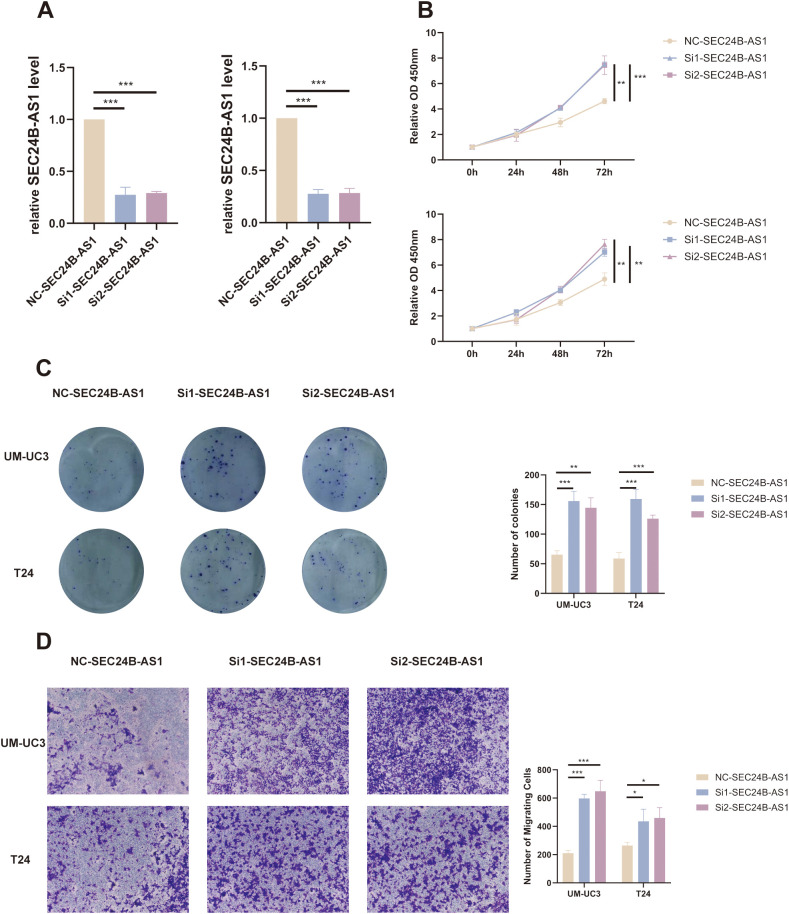
SEC24B-AS1 suppresses proliferation and migration of bladder cancer cells. **(A)** qRT-PCR analysis confirming the knockdown efficiency of SEC24B-AS1 in UM-UC3 and T24 cells following siRNA transfection. **(B)** CCK-8 assay results showing that silencing SEC24B-AS1 significantly enhances the proliferative ability of bladder cancer cells over time. **(C)** Representative images and quantification from the colony formation assay demonstrating that SEC24B-AS1 knockdown increases both the number and size of colonies compared with the control group. **(D)** Transwell migration assay demonstrating that SEC24B-AS1 depletion significantly increases the number of migrating cells compared with the control group. Statistical significance was determined using the independent Student’s t-test (n=3). *p < 0.05, **p < 0.01, ***p < 0.001.

To further confirm and strengthen the reliability of our migrasome-related lncRNA–based prognostic signature, a series of functional experiments were conducted focusing on SEC24B-AS1, one of the key components of the signature. Quantitative real-time PCR (qRT-PCR) analysis revealed that SEC24B-AS1 expression was markedly reduced following siRNA-mediated knockdown in UM-UC3 and T24 bladder cancer cell lines, indicating efficient silencing (**Figure 13A**). We next examined the role of SEC24B-AS1 in regulating cell proliferation. The CCK-8 assay demonstrated that depletion of SEC24B-AS1 significantly enhanced the proliferative capacity of tumor cells (**Figure 13B**). Consistently, the colony formation assay showed that SEC24B-AS1 knockdown increased the number and size of cell colonies, further supporting its growth-suppressive function (**Figure 13C**). To evaluate the impact of SEC24B-AS1 on cell migration, Transwell and wound-healing assays were performed. The Transwell assay revealed a remarkable increase in the number of migrating cells after SEC24B-AS1 silencing (**Figure 13D**).

**Figure 14:**
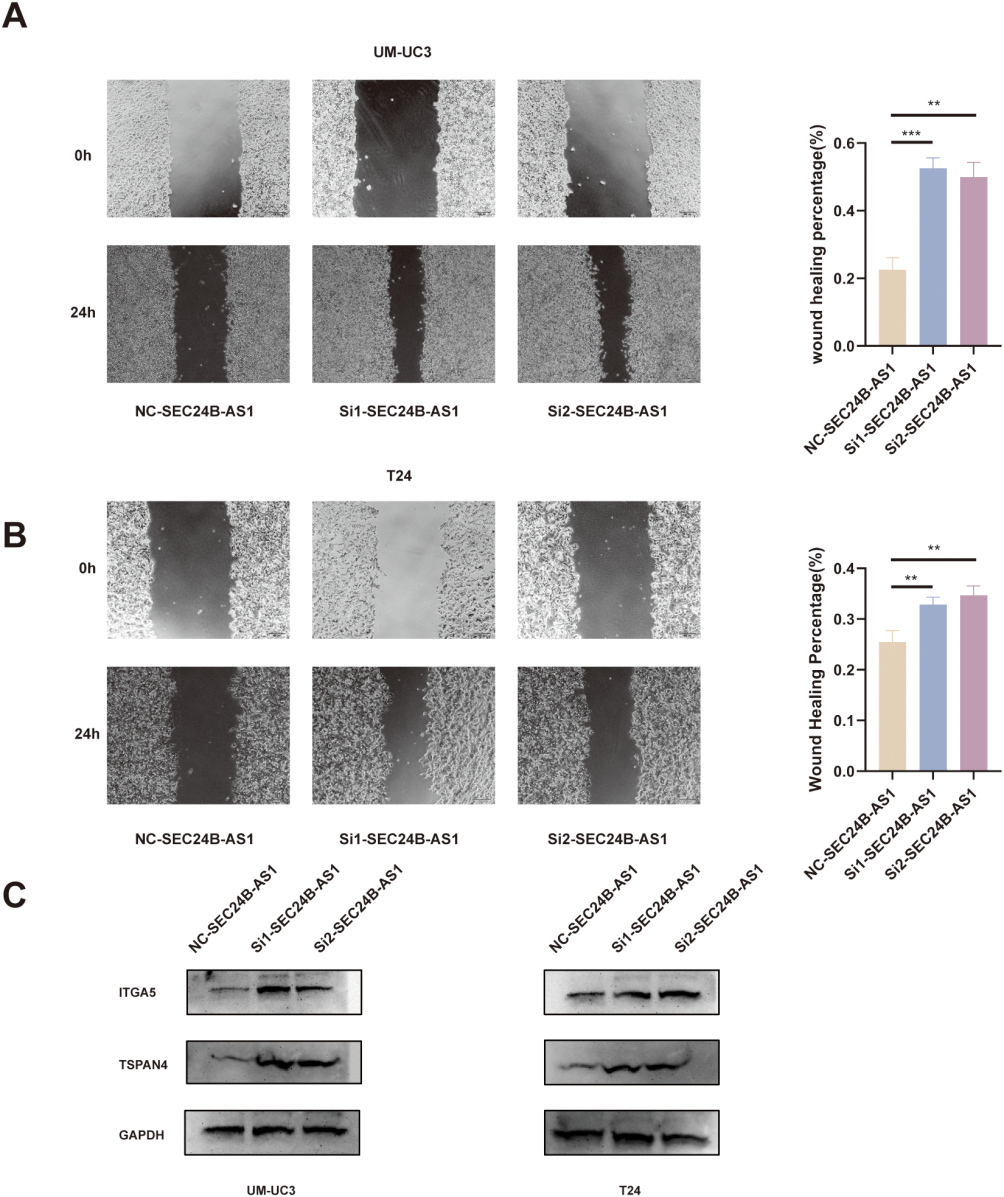
SEC24B-AS1 knockdown enhances the migratory ability of BLCA cells and increases the expression of migrasome markers. **(A, B)** Wound-healing assay showing that silencing SEC24B-AS1 markedly promotes cell migration, as evidenced by accelerated wound closure after 24 hours in UM-UC3 and T24 cells. Statistical significance was determined using the independent Student’s t-test (n=3). *p < 0.05, **p < 0.01, ***p < 0.001. **(C)** Knockdown of SEC24B-AS1 upregulated the expression of ITGA5 and TSPAN4.

Similarly, the wound-healing assay showed accelerated wound closure in the SEC24B-AS1 knockdown group compared with the control, indicating enhanced migratory ability (**Figures 14A, B**). Furthermore, we found that knockdown of SEC24B-AS1 significantly upregulated the expression levels of the migrasome markers ITGA5 and TSPAN4 (**Figure 14C**). Collectively, these findings suggest that SEC24B-AS1 acts as a potential suppressor of proliferation and migration in bladder cancer cells, highlighting its functional significance and reinforcing its biological relevance within the proposed prognostic signature.

## 4 Discussion

Migrasomes, recently identified as a type of extracellular vesicle formed during cell migration, have emerged as novel mediators of intercellular communication. They are known to carry bioactive molecules including proteins and RNAs, and play crucial roles in processes such as immune regulation, organ development, and pathological responses including tumor progression. A growing body of evidence suggests that migrasome-related genes may influence cancer cell behavior, including proliferation, invasion, and metastasis. However, their potential prognostic value in bladder cancer remains largely unexplored.

Based on a comprehensive literature review, we identified a panel of migrasome-associated genes, including TSPAN4, NDST1, PKD1, CPQ, PKD2, EPCIP, EOGT, TMX2-CTNND1, PIGK, ITGA5, and ITGB1. These genes were subsequently used as the foundation for screening migrasome-related long non-coding RNAs (lncRNAs). TSPAN4 promotes migrasome formation in retinal pigment epithelial (RPE) cells, enhancing their migration and proliferation via the TGF-β1/Smad2/3 signaling pathway, thereby contributing to PVR development (14). TSPAN4 contributes to tumor progression by promoting cancer cell proliferation and fostering an immunosuppressive microenvironment, particularly through M2 macrophage polarization and association with tumor heterogeneity and stemness (16). NDST1 maintains macrophage homeostasis by regulating heparan sulfate sulfation, thereby suppressing type I interferon signaling and limiting proinflammatory activation, foam cell formation, and atherogenesis (17). Ndst1-mediated N-sulfation of heparan sulfate is essential for maintaining proper podocyte structure and adhesion, with its loss leading to foot process effacement and abnormal glomerular architecture (18). PKD1 mutations exhibit adaptive functions in the liver by promoting regeneration and alleviating steatosis and glucose intolerance, without increasing the risk of malignant transformation (19). PKD1 acts as a neuroprotective, anti-apoptotic kinase that mitigates oxidative stress-induced neuronal injury by modulating apoptotic signaling pathways (20). The cryo-EM structure of the PKD1-PKD2 complex elucidates its noncanonical TRP channel architecture with a disrupted S6 helix and cation-blocking residues, providing mechanistic insights into ion permeability dysregulation and ADPKD pathogenesis driven by mutations in these genes (21). EOGT encodes an enzyme involved in the O-GlcNAcylation of extracellular EGF-domain-containing proteins, playing a role in epithelial-cell-matrix interactions (22). EOGT contributes to an immunosuppressive microenvironment in HCC by promoting T cell exhaustion and reducing CD8^+^ T cell infiltration, thereby associating with tumor progression and poor prognosis (23). EOGT promotes HCC cell proliferation and ferroptosis resistance by upregulating SLC7A11 through HEY1, contributing to poor prognosis and drug resistance (24). TMX2-CTNND1 is associated with schizophrenia susceptibility and may influence cognitive function by affecting cortical thickness in the right pars triangularis (25). PIGK is essential for neural development and Purkinje cell survival, with its deficiency leading to excessive unfolded protein response, apoptosis, and neurodevelopmental defects characteristic of GPIBD22 (26). PIGK is a key component of the GPI transamidase complex responsible for attaching GPI anchors to proteins, and its deficiency impairs GPI-anchored protein expression, leading to neurodevelopmental disorders including cerebellar atrophy and intellectual disability (27). ITGA5 promotes malignant behavior in TNBC by activating the FAK/PI3K/AKT signaling pathway, regulated by circRPPH1 via miR-326 sponging (28). ITGA5 promotes HCC progression and cancer stemness by transferring from stromal myofibroblasts to tumor cells via EVs, activating YES1 signaling, and contributing to poor prognosis and resistance to immunotherapy (29). ITGA5 marks a pro-inflammatory synovial fibroblast subset that promotes RA progression by driving TPH cell differentiation via TGF-β1 secretion and is associated with inflammation, multidrug resistance, and tissue remodeling (30). ITGB1 promotes oral cancer progression by enhancing cancer cell proliferation and cisplatin resistance, driven by circRNF13-mediated stabilization of its mRNA via m6A-dependent IGF2BP1 regulation (31). ITGB1, through CD44/CD29 clustering, activates Rac1 and promotes migration of AD-MSCs, contributing to cancer-associated fibroblast formation in a Muc5ac-dependent manner (32).

In this study, we developed and validated a novel prognostic model based on migrasome-related long non-coding RNAs (lncRNAs) in bladder cancer. Through rigorous bioinformatic analyses, we identified a set of lncRNAs significantly associated with patient survival and constructed a risk signature that effectively stratified patients into high- and low-risk groups across training, validation, and entire cohorts. The signature demonstrated strong predictive performance in both Kaplan-Meier survival analysis and ROC curve evaluation, outperforming conventional clinical features such as stage and grade. The migrasome-related lncRNA signature developed in this study not only demonstrated robust prognostic performance but also reflected distinct underlying biological features across different risk groups.

Immune infiltration patterns showed remarkable divergence between high- and low-risk subgroups. The high-risk group exhibited elevated stromal and immune scores, accompanied by increased infiltration of M2 macrophages. Conversely, the low-risk group demonstrated a higher proportion of Plasma cells, CD8+ T cells and Tregs. Furthermore, immune functional analysis revealed enhanced activity of immune-regulatory pathways—such as inflammation-promoting and check points—in high-risk patients. In terms of metabolic characteristics, gene set enrichment analysis highlighted the enrichment of drug metabolism cytochrome p450, linoleic acid metabolism, metabolism of xenobiotics by cytochrome p450, and retinol metabolism in the low-risk group. In contrast, the high-risk group was associated with increased activity in immune system pathways and extracellular matrix interactions, including chemokine signaling pathway, cytokine-cytokine receptor interaction, hematopoietic cell lineage, and focal adhesion. Somatic mutation profiles further emphasized the biological divergence. The high-risk group showed a higher tumor mutation burden (TMB) and frequent alterations in genes such as *TP53* and *TTN*. Moreover, patients with low TMB combined with high-risk scores exhibited the poorest survival outcomes. Supporting this, TIDE analysis revealed a significantly higher immune evasion potential in the high-risk cohort.

Finally, drug sensitivity analysis uncovered differential responsiveness to targeted agents. Low-risk patients showed greater sensitivity to compounds affecting cellular energy metabolism, intracellular signaling pathways and epigenetic regulation, such as Sorafenib and Nilotinib, while high-risk individuals responded more favorably to agents modulating apoptosis and immune pathways, including AZD7762 and Alpelisib. These findings offer insights into the biological underpinnings of the lncRNA signature and provide a rationale for tailored therapeutic strategies.

In recent years, numerous prognostic models based on long non-coding RNAs (lncRNAs) have been developed to predict outcomes in bladder cancer. These models have primarily focused on molecular features such as immune-related lncRNAs, ferroptosis-associated lncRNAs, or autophagy-related lncRNA signatures (33, 34). While these approaches have provided valuable prognostic insights, their biological mechanisms often remain unclear, and their applicability across multiple datasets is limited due to overfitting or lack of external validation.

Compared to these models, our study presents several notable innovations. First, to our knowledge, this is the first prognostic model derived from migrasome-related lncRNAs in bladder cancer. Migrasomes, as a novel type of extracellular vesicles involved in cell migration and intercellular communication, have only recently been implicated in tumor biology. By integrating migrasome biology with lncRNA expression profiles, our model not only offers prognostic value but also connects with an emerging mechanistic axis in cancer progression. Second, our signature demonstrated superior predictive performance compared to conventional clinical variables such as stage and grade, as evidenced by both time-dependent ROC analysis and Kaplan-Meier survival curves across training, validation, and entire cohorts. Importantly, the model maintained consistent stratification power, highlighting its robustness and generalizability. Third, unlike many previous models that lack in-depth functional exploration, we conducted comprehensive analyses including immune infiltration, metabolic pathway enrichment, mutational landscape, and drug sensitivity prediction. These efforts uncovered the biological underpinnings of the lncRNA signature, providing mechanistic insights that may guide precision therapy. Collectively, our study not only extends the current understanding of lncRNA-based prognosis in bladder cancer but also pioneers the incorporation of migrasome-associated regulatory networks into prognostic modeling. This provides a novel perspective for both biomarker development and the exploration of lncRNA functionality in the tumor microenvironment.

Despite the promising findings of our study, several limitations should be acknowledged. Firstly, our analysis was primarily based on the public TCGA transcriptomic dataset, and we lacked an independent external validation cohort. This is due to a technical limitation: some available public BLCA datasets with survival data are largely based on legacy microarray platforms, which exhibit incomplete probe coverage for the core novel lncRNAs identified in our RNA-seq-based signature. Therefore, future prospective, multi-center studies utilizing deep RNA sequencing are essential to fully validate the clinical generalizability of this model. To compensate for this limitation and mitigate the risk of statistical overfitting, we rigorously conducted *in vitro* biological functional assays. While external cohorts primarily test the mathematical reproducibility of a model, our biological validations confirm the true functional relevance of the identified lncRNAs in driving cancer progression, thereby providing a robust and higher-order justification for the reliability of our signature. Secondly, due to incomplete clinical information in the TCGA dataset, certain key factors such as treatment history, recurrence status, and comorbidities could not be included in the prognostic modeling. Thirdly, although our bioinformatic analyses strongly suggest the metabolic and immunological involvement of the identified lncRNA signature, direct experimental validation of the specific immunomodulatory mechanisms—such as evaluating macrophage polarization or T-cell infiltration via *in vitro* co*-*culture assays—is still required in future studies. Future studies will aim to incorporate multi-center clinical data to confirm the clinical and biological relevance of the signature.

## 5 Conclusion

In conclusion, we developed a novel prognostic signature based on migrasome-related lncRNAs that effectively stratifies bladder cancer patients by survival risk. This signature demonstrates robust predictive performance and is associated with distinct immune landscapes, metabolic activities, mutation burdens, and drug sensitivities. Our findings not only provide insights into the biological roles of migrasome-related lncRNAs but also offer potential avenues for personalized therapy in bladder cancer.

## Data Availability

The dataset TCGA-BLCA utilized in this research is publicly accessible through the GDC Data Portal (https://portal.gdc.cancer.gov/). The single-cell RNA sequencing datasets can be found in the Gene Expression Omnibus (GEO) database under the accession numbers GSE267718 (including samples GSM8273655, GSM8273657, GSM8273661, GSM8273665, GSM8273672, and GSM8273675) and GSE130001 (including samples GSM3729178 and GSM3729179).
