# Supplementary material for: Identification of a migrasome-related lncRNA signature and its prognostic and immunological role in bladder cancer
Source: Front Immunol. 2026 Apr 16;17:1763443. doi: 10.3389/fimmu.2026.1763443 (PMC13128627; doi:10.3389/fimmu.2026.1763443)
Supplement: Supplementary file 1 [file DataSheet1.pdf]

Supplementary Material

1 Supplementary Figures

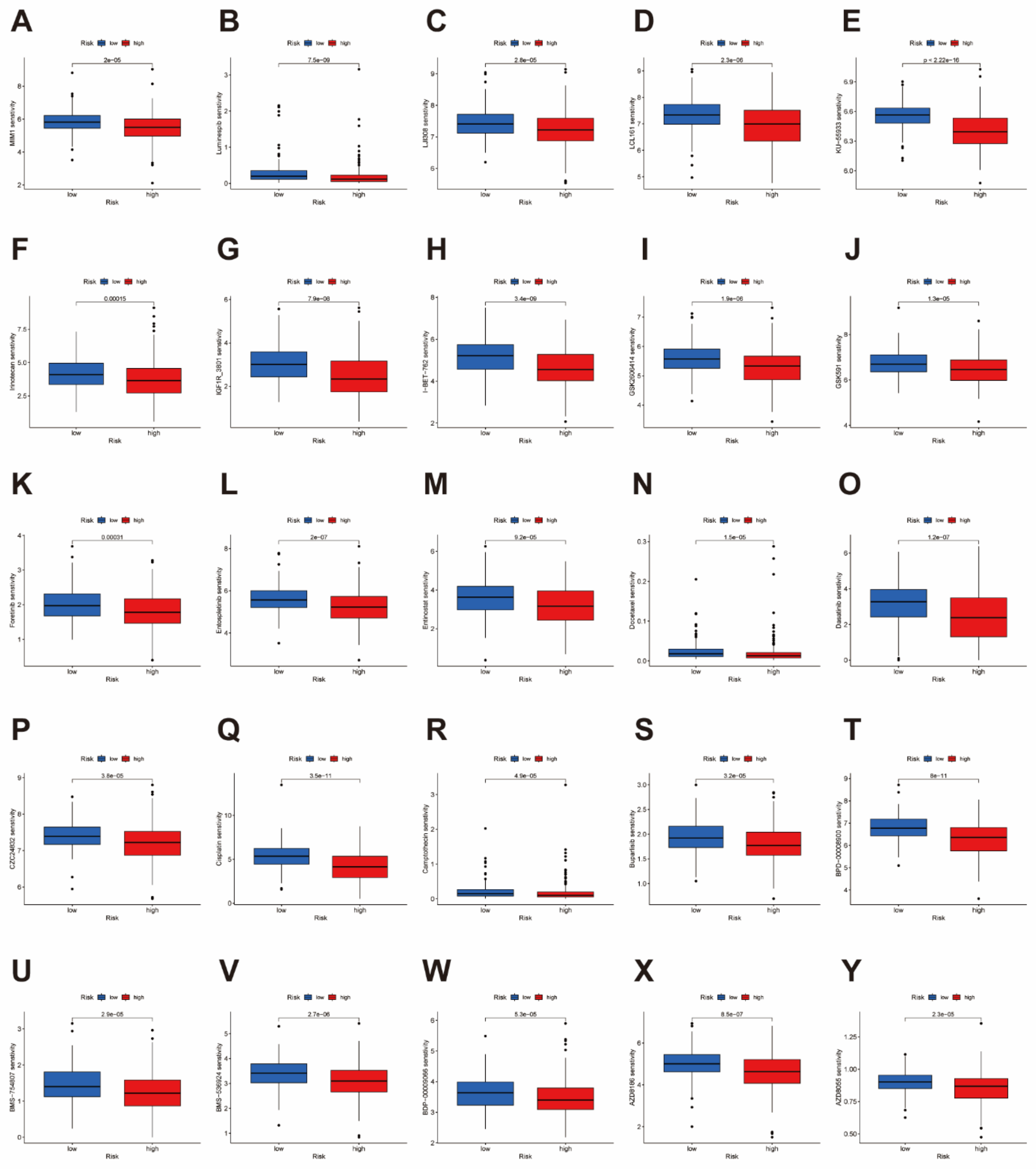

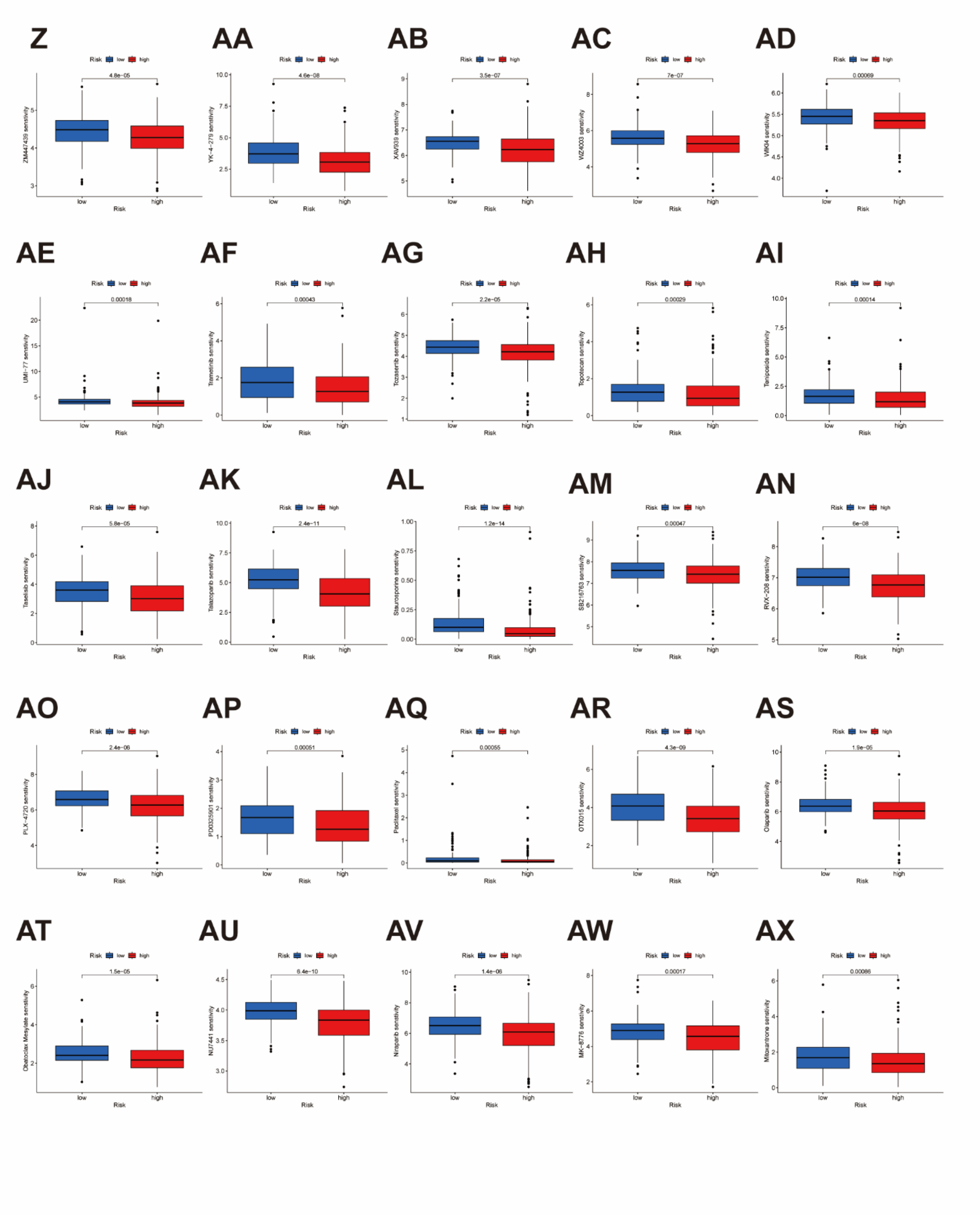

Supplementary Figure S1 (A-AX) Drug sensitivity analysis revealed the drugs to be predicted as more sensitive in the BLCA high-risk group.

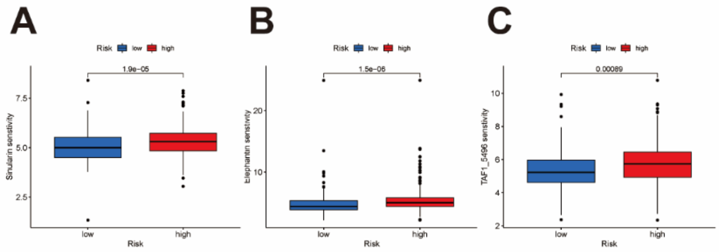

Supplementary Figure S2 (A-C) Drug sensitivity analysis revealed the following drugs to be predicted as more sensitive in the BLCA low-risk group.

2 Supplementary Tables

	Primers or siRNA sequence (5′-3′)
GAPDH-F	5′- ACAACTTTGGTATCGTGGAAGG-3′
GAPDH-R	5′- GCCATCACGCCACAGTTTC-3′
SEC24B-AS1-R	5′- CCTTGGACAACTGAACGCCT-3′
SEC24B-AS1-F	5′- CGTGGTGAAACTCAACTGGA-3′
si SEC24B-AS1#1 Sense strand	5′- CAGAUUUAUUACAUUUAUAAUTT-3′
si SEC24B-AS1#1 Antisense strand	5′- AUUAUAAAUGUAAUAAAUCUGTT-3′
si SEC24B-AS1#2 Sense strand	5′- CUACUGACUUGUAUUUGUAUUTT-3′
si SEC24B-AS1#2 Antisense strand	5′- AAUACAAAUACAAGUCAGUAGTT-3′

Supplementary Table 1 The sequences of qRT-PCR primers and siRNA

Covariates	Type	Total	Test	Train	Pvalue
Age	<=65	159(39.36%)	78(38.61%)	81(40.1%)	0.8386
Age	>65	245(60.64%)	124(61.39%)	121(59.9%)	
Gender	FEMALE	106(26.24%)	46(22.77%)	60(29.7%)	0.1415
Gender	MALE	298(73.76%)	156(77.23%)	142(70.3%)	
Grade	High Grade	381(94.31%)	193(95.54%)	188(93.07%)	0.792
Grade	Low Grade	20(4.95%)	9(4.46%)	11(5.45%)	
Grade	unknow	3(0.74%)	0(0%)	3(1.49%)	
Stage	Stage I	2(0.5%)	0(0%)	2(0.99%)	0.0933
Stage	Stage II	128(31.68%)	57(28.22%)	71(35.15%)	
Stage	Stage III	140(34.65%)	80(39.6%)	60(29.7%)	
Stage	Stage IV	132(32.67%)	65(32.18%)	67(33.17%)	
Stage	unknow	2(0.5%)	0(0%)	2(0.99%)	
T	T0	1(0.25%)	1(0.5%)	0(0%)	0.0863
T	T1	3(0.74%)	0(0%)	3(1.49%)	
T	T2	117(28.96%)	53(26.24%)	64(31.68%)	
T	T3	193(47.77%)	108(53.47%)	85(42.08%)	
T	T4	57(14.11%)	26(12.87%)	31(15.35%)	
T	unknow	33(8.17%)	14(6.93%)	19(9.41%)	
M	M0	194(48.02%)	99(49.01%)	95(47.03%)	0.2213
M	M1	11(2.72%)	3(1.49%)	8(3.96%)	
M	unknow	199(49.26%)	100(49.5%)	99(49.01%)	
N	N0	235(58.17%)	126(62.38%)	109(53.96%)	0.5643
N	N1	46(11.39%)	25(12.38%)	21(10.4%)	
N	N2	75(18.56%)	35(17.33%)	40(19.8%)	
N	N3	6(1.49%)	2(0.99%)	4(1.98%)	
N	unknow	42(10.4%)	14(6.93%)	28(13.86%)	

Supplementary Table S2 Clinical characteristics between groups and p-values for differences between the training and validation cohorts.
